# Crystal structure of fatty acid thioesterase A bound by 129 fragments provides diverse development opportunities

**DOI:** 10.1002/ps.70199

**Published:** 2025-09-12

**Authors:** Ekaterina Kot, Matteo Paolo Ferla, Patricia Helen Hollinshead, Charles William Ernest Tomlinson, Daren Fearon, Jasmin Cara Aschenbrenner, Lizbé Koekemoer, Max Winokan, Michael Fairhead, Xiaomin Ni, Rod Chalk, Katherine Sara England, Laura Ortega Varga, Mark Greer Montgomery, Nicholas Phillip Mulholland, Frank von Delft

**Affiliations:** ^1^ Centre for Medicines Discovery Oxford UK; ^2^ Syngenta Crop Protection, Jealott's Hill International Research Centre Bracknell UK; ^3^ Diamond Light Source Ltd, Harwell Science and Innovation Campus Didcot UK; ^4^ Research Complex at Harwell, Harwell Science and Innovation Campus Didcot UK; ^5^ Alzheimer's Research UK Oxford Drug Discovery Institute, Centre for Medicines Discovery, Nuffield Department of Medicine Oxford UK; ^6^ Department of Biochemistry University of Johannesburg Aukland Park South Africa

**Keywords:** crystallographic fragment screening, fatty acid thioesterase A, acyl‐acyl carrier protein thioesterase, structure‐based discovery, herbicide, fragment merging

## Abstract

**BACKGROUND:**

In order to alleviate the growing issue of herbicide resistance, diversification of the herbicide portfolio is necessary. A promising yet underutilized mode‐of‐action is the inhibition of fatty acid thioesterases (FATs), which terminate *de novo* fatty acid (FA) biosynthesis by releasing FAs from acyl carrier protein (ACP) cofactors. These enzymes impact plant growth and sterility by determining the amount and length of FAs present. In this study we report a crystallographic fragment screening approach for the identification of novel chemical matter targeting FATs.

**RESULTS:**

We have solved the crystal structure of *Arabidopsis thaliana* FatA to 1.5 Å and conducted a crystallographic fragment screen which identified 129 unique fragments bound in 141 different poses. Ten fragments demonstrated on‐scale potency, two of these exploiting different interactions to known herbicides. Elaboration of one of the fragments resulted in an improvement of affinity from ~20 μm to ~90 nm *K*_D_. Finally, superposition of our crystal structures revealed that some fragments exploit large conformational changes in the substrate binding site.

**CONCLUSION:**

We have fully enabled FatA as a target for rapid, rational hit‐to‐lead development, with robust structural, biophysical and biochemical assays. We provide a set of fragment hits which represent diverse, novel scaffolds that both recapitulate interactions made by current herbicides, and also target novel regions within the active and dimer sites. Our fragments can be readily merged and allow for effective catalogue‐based structure–activity relationship (SAR) exploration. Together these data will accelerate the development of novel, alternative herbicides to combat herbicide resistance. © 2025 The Author(s). *Pest Management Science* published by John Wiley & Sons Ltd on behalf of Society of Chemical Industry.

## Graphical abstract

Crystallographic fragment screening of *A. thaliana* FatA yielded 141 hits and along with orthogonal assays, this enables the target for rapid development of novel chemical matter to the currently available herbicides.

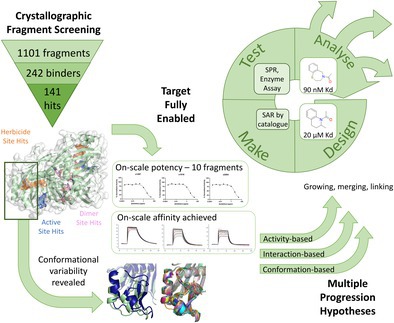

## 1 INTRODUCTION

Herbicide resistance is a growing issue threatening global food security. Of the 31 biochemical processes targeted by herbicides, resistance has been reported against 21, involving 168 different herbicides.^1^ Herbicide resistance can be mitigated by sequential or combinatorial use of herbicides with different mechanisms of controlling the plants, known as modes‐of‐action (MoAs). In order to facilitate this approach, herbicides must be developed against novel or underutilized molecular targets.

Fatty acid thioesterases (FATs) are a promising herbicidal target. Though the herbicide cinmethylin had been on the market for decades, its MoA was only elucidated in 2018, when it was identified by BASF as a FAT inhibitor.^2^ Since then, several other herbicides—including methiozolin, bromobutide, cumyluron and tebutam—have been retrospectively classified under the same MoA.^3^, ^4^, ^5^ As of 2025, the Herbicide Resistance Action Committee (HRAC) has placed FAT inhibitors into Group 30.^3^ Inhibition of FATs offers an attractive MoA owing to their absence from Animalia and a subsequently lower risk of toxicity. Moreover, FAT herbicides offer an effective way to control herbicide‐resistant grasses.^6^ In 2023, a new chemical motif, 1,8‐naphthyridine, was described by Bayer.^7^ Several subsequent publications have employed scaffold‐hopping approaches to access novel inhibitors based either on 1,8‐naphthyridine or existing herbicides such as methiozolin.^7^, ^8^, ^9^, ^10^, ^11^

**Figure 1:**
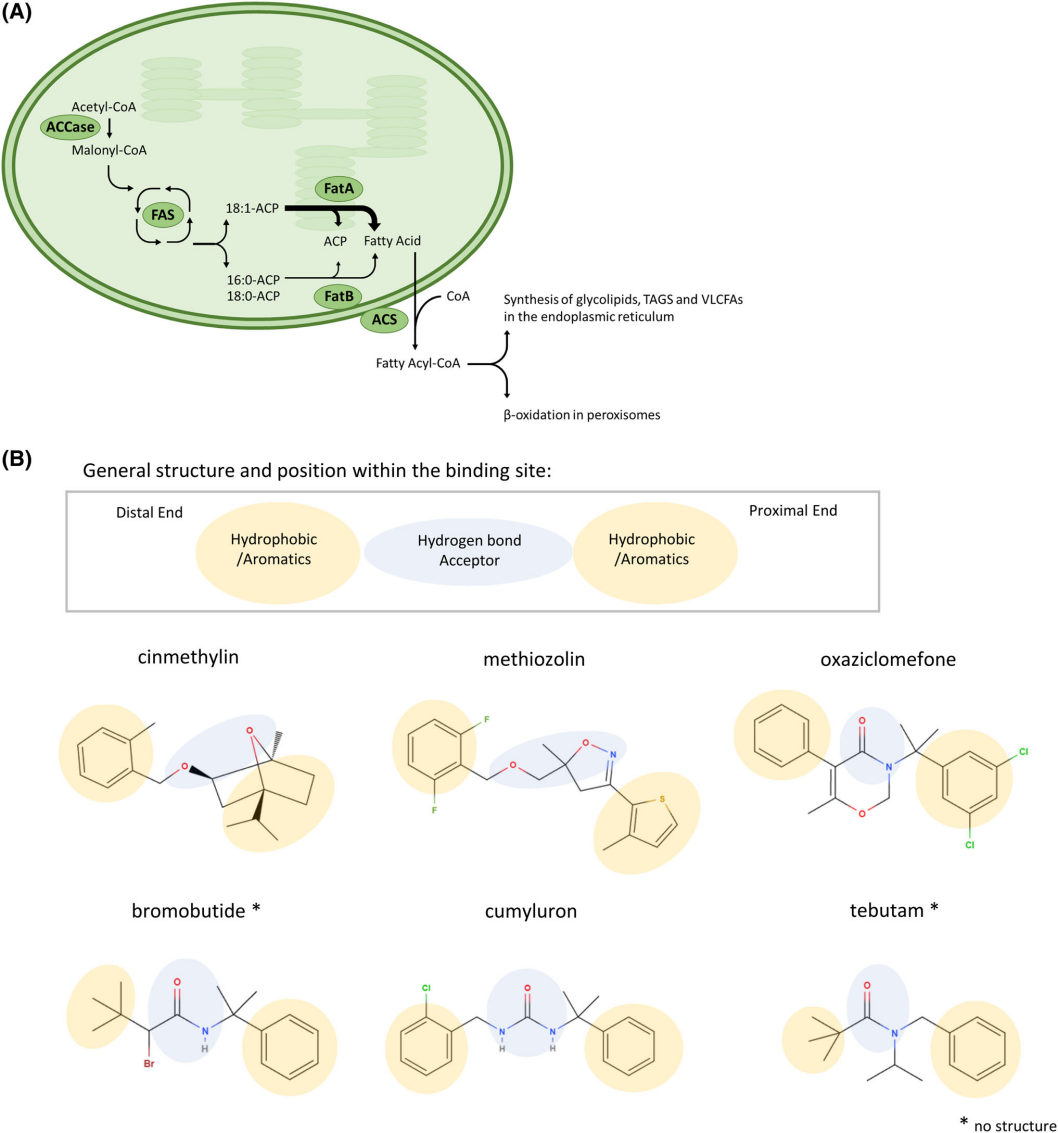
Biological pathway of FatA and its current inhibitors. (A) A simplified overview of fatty acid metabolism in the plastids of plants. (B) Chemical structures of currently recognized herbicides targeting FatA highlighting similarity in their general pharmacophoric profile. Distal and Proximal Ends refer to orientation within the herbicide binding pocket based on their structures or estimates where no structure was available. CoA, coenzyme A; ACCase, acetyl‐coenzyme A carboxylase; FAS, fatty acid synthase; Fat, fatty acid thioesterase; ACP, acyl carrier protein; ACS, acyl‐CoA synthetase; TAGS, triacylglycerols; VLCFAs, very‐long chain fatty acids.

Nonetheless, the current FAT herbicides share a highly similar pharmacophore [Fig. 1(B)] and where structural information is available, they all bind in the same binding site and interact with a similar set of residues [Fig. 4(A)–(C)].^5^ This increases the risk of cross‐resistance. Reduced sensitivity towards cinmethylin has already been identified in blackgrass (*Alopecurus myosuroides*) with nontarget specific resistance to HRAC Group 1 and 3 herbicides, albeit when exposed to levels below the field‐rate dose.^12^ The current herbicides also can suffer from a lack of crop selectivity, soil‐dependent variability in efficacy and possess suboptimal physical properties such as high volatility (cinmethylin) and low water solubility.^4^, ^13^, ^14^, ^15^, ^16^ Additional chemotypes could present opportunities to improve on these properties.

FATs are involved in the *de novo* biosynthesis of fatty acids (FAs) in higher plants. Located in the plastids, they terminate FA elongation by catalyzing the cleavage of the thioester bond between the FA and an acyl carrier protein (ACP) cofactor [Fig. 1(A)]. They play a key role in determining the quantity and type of FAs produced by plants, and in turn, affect plant growth and sterility.^17^, ^18^ There are two families of FATs: FatAs which have a preference for unsaturated oleic acid (C18:1) and FatBs which have broader specificity and a preference for saturated C18:0 and C16:0 chains.^19^, ^20^ The first published crystal structure of a plant thioesterase was FatB from *Umbellularia californica*, yet recently three FatA structures from *Lemna aequinoctialis* bound to spirolactam ligands have been released.^8^, ^21^

Because knockout of both enzymes is necessary for a lethal phenotype, it is thought that current herbicides must be dual inhibitors of both FatA and FatB.^2^ However, FatA has been more extensively characterized as a result of the simplicity of its purification and crystallization, and its inhibition *in vitro* correlating well with *in vivo* outcomes.^5^ We have therefore selected *Arabidopsis thaliana* FatA as a protein target for developing novel chemical entities, while keeping in mind that effective inhibitors will ultimately need to target both FAT families.

Crystallographic fragment screening is a powerful method for discovering novel starting points for inhibitor design. Because the size of the fragments is so small (usually <250 Da), they explore the chemical space more efficiently and are more likely to be complementary to the target than the larger, more complex compound libraries used in traditional high‐throughput screening.^22^, ^23^ Using the high‐throughput fragment screening facilities of XChem at Diamond Light Source (DLS), thousands of fragments can be screened in a week to explore the molecular recognition capabilities of a protein target.^24^, ^25^ Several such campaigns have led to hit discoveries and hit‐to‐lead compound progression.^26^, ^27^, ^28^, ^29^ The agrochemical field has been slow to adopt routine fragment‐based screening because of the greater accessibility of *in vivo* phenotypic screening at the early stages of development when compared to pharmaceutical research. However, when trying to develop inhibitors with novel chemical scaffolds or orthogonal mechanisms of binding, starting from smaller building blocks can be highly beneficial, hence our use of fragments in this campaign.

In this study we apply crystallographic fragment screening to find starting points for rational discovery of novel inhibitors with alternative binding modes to the currently available herbicides. We have built on in‐house work from Syngenta to establish a robust crystallization system suitable for an XChem campaign. We report a 1.5 Å crystal structure of *A. thaliana* FatA as well as a set of 129 fragment‐bound structures, which provide 141 different starting points for future inhibitor development. Ten fragments already show potency in the double digit micromolar range in an enzymatic assay, with the top fragment having a median inhibitory concentration (IC_50_) of <15 μm. Three binding sites of interest have been identified, each occupied by a diverse set of chemical scaffolds. Comparison of fragment‐bound structures revealed that some ligands can exploit large conformational changes within FatA and that the N‐terminal portion responsible for substrate binding displays much greater flexibility than the catalytic C‐terminal half. As a result of this campaign, several diverse follow‐up opportunities have emerged.

## 2 RESULTS

### 2.1 The crystal structure of FatA reveals a dimer and adopts the same fold as FatB

A crystal structure of FatA from *A. thaliana*, a widely used model organism, was obtained using the protocol previously established by Syngenta (Syngenta, personal communication). The construct includes a truncation at the N‐terminus to remove the chloroplast transit peptide and a noncleavable His‐tag at the C‐terminus. The previously established crystallization condition consisted of MES and ammonium sulfate, which yielded consistent crystals that appeared after 1 day and diffracted to ≈1.7 Å. The structure was solved by molecular replacement using a crystal structure provided by Syngenta (Syngenta, personal communications).

Automatic data reduction pipelines at Diamond often attributed each crystal to both the I422 and the I222 space groups. Because the former results in a monomer within the asymmetric unit, we chose to process all of our data as I222. Processing the two protein chains separately allowed us to distinguish between the occasional conformational variability between the two chains, which is present in only some of the datasets. Moreover, we believe that the crystallographic dimer we observe is in fact physiological as native mass spectroscopy data shows a dimer to be the predominant species (Supporting information Fig. S1). Our crystallographic dimer also maps faithfully onto the published FatB structure, which was shown to be physiological through mutations at the dimer interface.^21^

**Figure 2:**
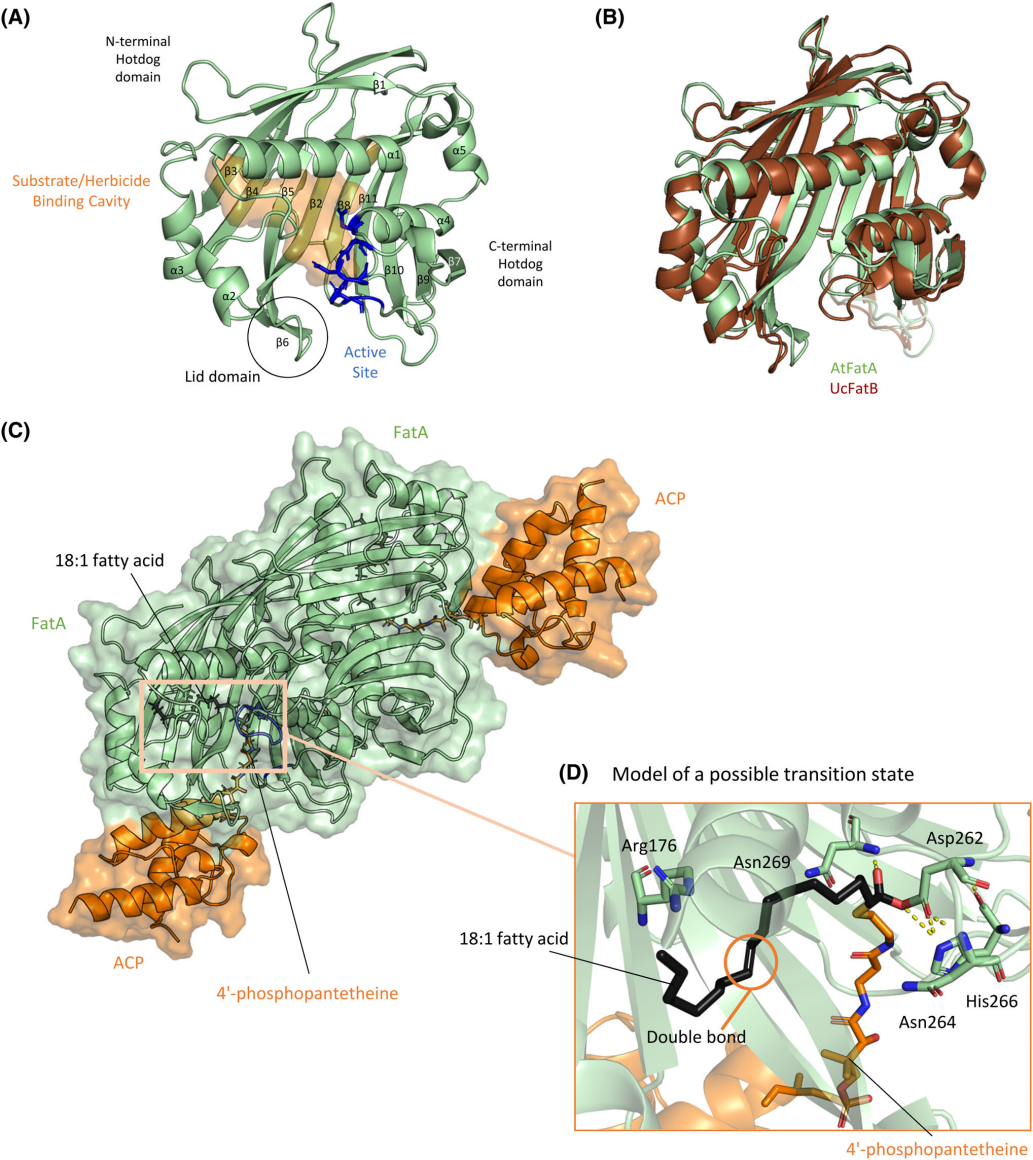
Biological assembly of FatA can be modelled using the crystal structure. (A) Crystal structure of *Arabidopsis thaliana* FatA. The substrate and herbicide binding cavity is shown in orange. Catalytic residues (Asp262, Asn264, His266, Asn269 and Glu300) are shown as blue sticks. *α*‐helices and *β*‐strands which make up two tandem Hotdog folds are labelled. (B) Overlay of FatA from *A. thaliana* (AtFatA, PDB: 9HRQ) and FatB from *Umbellularia californica* (UcFatB, PDB: 5X04) crystal structures. AtFatA is shown in green; UcFatB is shown in brown. (C) Predicted model of ACP (shown in orange) bound to the crystallographic AtFatA dimer (shown in green) with an 18:1 fatty acid (shown in black) placed into the predicted substrate binding cavity. (D) Zoomed‐in view of a possible transition state. The 18:1 fatty acid (shown in black) is covalently bound to the catalytic Asp262, which is coordinated by Asn264 and His266 (H‐bonds shown by yellow dashed lines). Asn269 helps stabilize the intermediate. The double bond of the 18:1 fatty acid is circled in orange to highlight its proximity to Arg176.

FatA from *A. thaliana* adopted the same fold as FatB from *U. californica* [Fig. 2(B)]^21^; the two possess a 42.4% sequence identity. The dimer contains two chains, each chain forming two tandem Hotdog folds which are connected by a long linker through the back of the *β*‐sheet [Fig. 2(A)]. The N‐terminal half forms the substrate‐binding cavity access to which is granted by the lid domain, meanwhile the C‐terminal half contains the active site.^21^ Residues within the previously proposed catalytic centre—Asp262, His266, Glu300, Asn264 and Asn269—are located proximal to each other and in a way that does not contradict the possibility of their involvement.^21^, ^30^

Within the herbicide binding site of FatA, we often observed a well‐resolved density of an unidentified ligand [Fig. 5(F)]. In view of the large number of hits obtained during the fragment screen, we did not attempt identification of this ligand. We postulate this may be a metabolite from the *E. coli* expression host because it does not resemble any component of the protein buffer or the crystallization condition. However, upon soaking with DMSO as part of the solvent test, the density disappeared, thus we went ahead with a crystallographic fragment screen. An example of a structure containing the unidentified ligand, x0045, has been deposited in the PDB under ID 9HRQ, whereas the *apo* structure of FatA was deposited under ID 9HRR.

### 2.2 A model of FatA's biological assembly corroborates the enzymatic mechanism

The FatA–ACP complex was modelled using ColabFold and the FA was positioned based on the related *E. coli* FabA–AcpP complex structure crosslinked to a substrate mimic 1R3 [Fig. 2(C)].^31^ To model the ligand, we super‐positioned the FabA–AcpP structure (PDB ID: 4KEH) on our FatA–ACP complex, copied over ligand 1R3, modified it to resemble the C18:1 and energy‐minimized the structure with strong constraints to enforce poised catalytic binding.

The substrate was modelled as a possible tetrahedral transition state where it is covalently bound to Asp262 [Fig. 2(D)]. We observe coordination of the Asp262 sidechain oxygen (O) by the nitrogen (N) atoms from the backbone of Asn264 and the sidechain of His266. This supports the catalytic mechanism proposed by Feng *et al*. although they proposed that it is the backbone, not the sidechain, of His266 which would hydrogen (H)‐bond with Asp262.^21^ We also observe the backbone amide of Asn269 stabilizing the negative charge on the deprotonated O of the intermediate, thereby forming an oxyanion hole.

The long FA fits well into the internal hydrophobic cavity of FatA, which is largely occluded from the outside with the access point between the lid domain and the active site. The double bond within the FA lines up to be within 4 Å of NE of Arg176, whose presence was perplexing within such a hydrophobic cavity. However, because this arginine also is present in FatB it is unlikely to contribute to the specificity of FatA towards C18:1.

### 2.3 One hundred and twenty‐nine fragments were found to bind FatA during a crystallographic fragment screen

A crystallographic fragment screen was conducted at Diamond's XChem facility. Two crystallization conditions were tested in a small 100‐fragment pre‐screen: the one used to obtain the *apo* structure (consisting of MES and ammonium sulfate) as well as one using sodium cacodylate as a buffer instead. No improvement in hit rate and crystal survivability was observed with cacodylate, so the main screen was conducted with MES as the crystallization buffer; however, hits unique to each condition were observed (see Data S2).

For the main screen, two libraries (Enamine Essential Fragments and DSi‐Poised^32^) were used yielding a total of 1101 compounds. There was a 21% attrition rate composed of soaking failures where the crystal either could not be mounted after soaking because of its deterioration, or diffraction failures where the successfully mounted crystals did not diffract.

**Figure 3:**
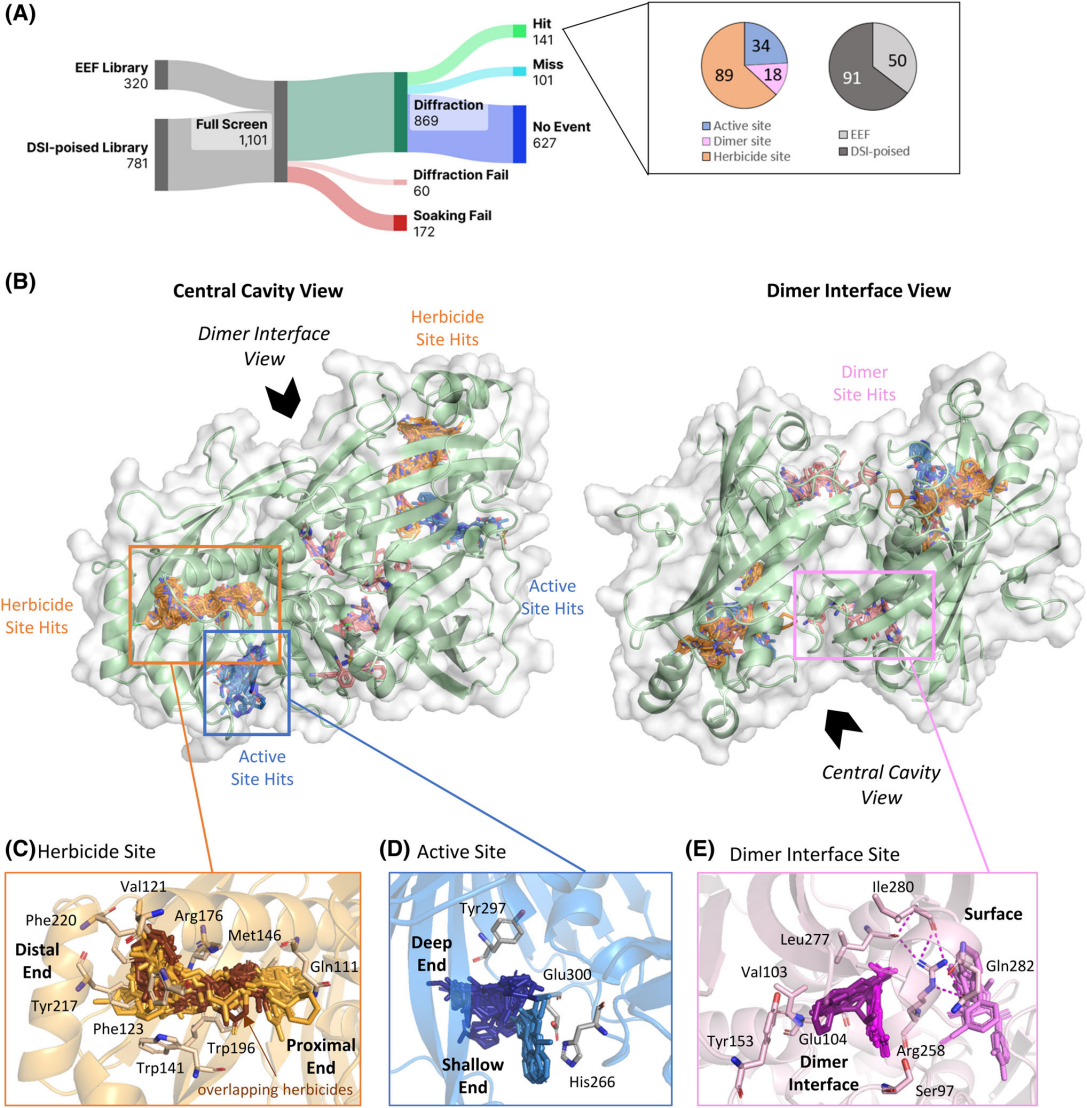
Fragments thoroughly sample the three binding sites of interest. (A) Sankey diagram of the crystallographic fragment screen of FatA with pie charts showing which libraries the fragments originated from and the distribution of hits across the sites of interest. EEF, Enamine Essential Fragment; DSI‐poised, Diamond‐SGC‐iNEXT poised. Sankey diagram produced by SankeyArt.com. (B) Structure of the FatA dimer bound by all fragment hits. Close‐ups of the (C) herbicide (D) active and (E) dimer interface sites showing frequently interacting residues. Where a site has been split into groups, these groups are labelled. For the herbicide binding site, fragment atoms which are within 1 Å of cinmethylin, methiozolin or oxaziclomefone are coloured brown to show the additional binding space (atoms shown in orange) explored by our fragments.

Subsequent processing with the PanDDA algorithm identified binding events in 242 datasets. We predetermined three sites of interest: the herbicide binding site—based on the structures of FatA bound by commercial herbicides; the active site—based on the position of residues previously shown to be important for catalysis; and the dimer site—located at the interface between the two protein chains chosen because formation of a dimer has been shown to be necessary for catalytic activity of plant FATs [Fig. 3(B)].^5^, ^21^, ^30^ Targeting the dimer interface carries the risk that binders either activate the enzyme or destroy the crystals and hence any readout of the binding pose. The number of fragments binding at the herbicide, active and dimer binding sites was 89, 34 and 18, respectively. This mirrors the relative sizes of these binding sites.

One hundred and twenty‐nine structures contained fragments bound to sites of interest and thus were processed further, giving rise to 141 individual hits [Fig. 3(A)]. The number of hits (141) is greater than the number of datasets (129) because the fragments which bound simultaneously at different sites within the same crystal constitute different starting points for further development and thus were counted separately. Fragments bound outside the sites of interest were termed ‘misses’ and not processed further.

There was no major difference in fragment binding to the two protomers in the asymmetric unit [Fig. S2; Data S2]. Of the 141 hits: 121 bound with same pose to both chains of FatA; 17 retained the binding pose, but produced ligand electron density of differential clarity suggesting differential occupancy of the two chains; and only three bound to only one protomer.

All fragment bound structures were deposited in the PDB under the group deposition ID G_1002328. Data on the full fragment screen, fragment hits and crystallographic information can be found in Data S2. Chemical structures of the fragment hits are available in Data S1.

#### 2.3.1 Fragments bound at the same site as commercial herbicides recapitulate key interactions as well as explore novel areas of the cavity

**Figure 4:**
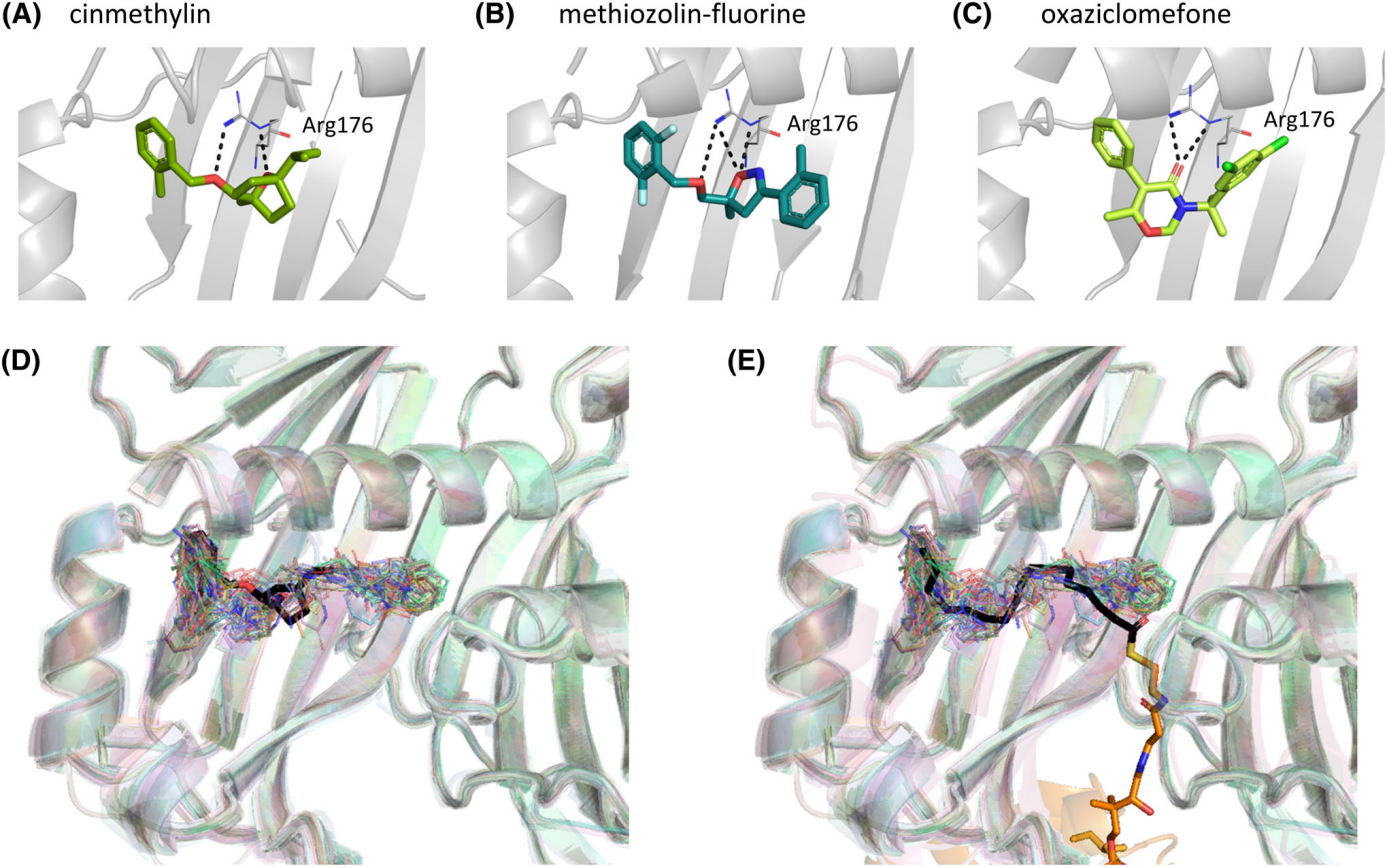
Fragments bound at the herbicide site overlap with commercial herbicides and the natural substrate. Crystal structures showing herbicides (A) cinmethylin (PDB: 9GRR), (B) methiozolin analogue methiozolin‐fluorine (PDB: 9HMT) and (C) oxaziclomefone (PDB: 9GS1) bound at the herbicide binding site and H‐bonding with Arg176. An overlay of all hit‐bound structures of FatA showing all fragments which bound at the herbicide binding site aligned with (D) a crystal structure of cinmethylin and (E) modelled structure of 18:1‐ACP.

The herbicide binding site was identified based on the fact that known commercial herbicides cinmethylin, methiozolin and oxazioclomefone bind here in a similar way [Fig. 4(A)–(C)]. Moreover, this is the same site that our ACP‐bound model suggests the natural substrate occupies during catalysis, making it likely that inhibitors bound here act by sterically excluding the substrate. We saw 89 fragments binding here. Because the cavity is quite large, the side closest to the active‐site was termed ‘proximal’ whereas the end of the binding pocket was termed ‘distal’ [Fig. 3(C)].

The key defining residue of this herbicide site is Arg176 which H‐bonded to all the known FATs inhibitors and also aligns with the double bond of the C18:1 substrate in our model. FATs herbicides also form *π*–*π* and hydrophobic interactions with Val121, Phe123, Tyr217, Phe220, Trp141 and Trp196 via their hydrophobic motifs on either side of the H‐bond acceptor.

We therefore compared the binding poses of our 89 fragments to that of the herbicides and the natural substrate [Fig. 4(D),(E)]. All of the interactions made by current herbicides were recapitulated by subsets of our fragments. Tyr217 and Phe123 were especially frequent sites of interaction with aromatic motifs of fragments frequently observed overlaying the aromatic portion of the herbicides (Fig. S3). However, our fragments explored a larger proportion of the substrate binding cavity, especially the proximal half. Gln111 and Met146 located here were especially popular hydrophobic interactors. Gln111 also frequently formed H‐bonds via its side‐chain such as with x1285 [Fig. S4(A)].

#### 2.3.2 Some fragments bound at the active site make direct interactions with the catalytic histidine

The active site is composed of four loops. Two loops formed of residues 266–269 and 298–300 are part of the C‐terminal half and contain the residues shown to be involved in catalysis. The other two loops are from the N‐terminal half: the tip of the 122–132 loop and residues 197–206 which form the lid domain. Val142 which is located on the end of a *β*‐strand nearby also forms some interactions with the fragments.

The 34 fragments bound at the active site were split into two groups: the ‘deep’ half bound between *β*2 and *β*8 strands and the ‘shallow’ half bound closer to the active site loops [Fig. 3(D)]. Fragments in the deep half commonly interacted with Tyr297, which, while noncatalytic, is still proximal to the active site (Fig. S3). It formed both hydrophobic interactions and H‐bonds via its backbone O, for example, to x1891 [Fig. S4(B)].

Within the shallow half of the catalytic residues, His266 and Asn269 make the most frequent interactions with the fragments, whereas Asn264 and Asp262 were largely uninvolved (Fig. S3). Because Glu300 has weak density, the reliability of interaction analysis is reduced. His266 was observed interacting via *π*‐stacking with aromatic motifs of five unique fragments, all of which contain two heterocyclic rings, fused in most (4/5) cases (Fig. S5).

#### 2.3.3 Fragments bound at the dimer interface cluster around an arginine involved in dimer interactions

The dimer site was bound by 18 fragments, most of which were found within the dimer interface near Arg258 and a few closer to the exterior surface [Fig. 3(B),(E)]. Arg258 previously has been shown to be key in maintaining the dimer interface via an H‐bond network.^21^ In the case of FatA, Arg258 forms H‐bonds with backbone Os of Leu277 and Ile280, and the side‐chain of Gln282 [Fig. 3(E)]. Several of our fragments interact directly with this network: x1231 and x1236 bind Arg258 via *π*–cation interaction and an H‐bond, respectively [Fig. S4(C),(D)].

Those that bind within the dimer interface pocket do so in two groups which are at a 60° to each other but overlap on one end and can be distinguished by their pattern of interactions [Fig. 3(E)]. Those closer to the surface can form H‐bonds with Ser97 such as x1211 [Fig. S4(E)], whereas the more buried group commonly interact with Val103 either hydrophobically via its side chain or form an H‐bond with its backbone NH. Interactions with Tyr153 also are exclusive to this group and highly versatile; we observed H‐bonding, *π*‐stacking and hydrophobic interactions depending on the fragment bound. At the point where both groups overlap, both sets of fragments can form H‐bonds with the side chain of Glu104.

### 2.4 Binding of some ligands induces large, asymmetric movement

**Figure 5:**
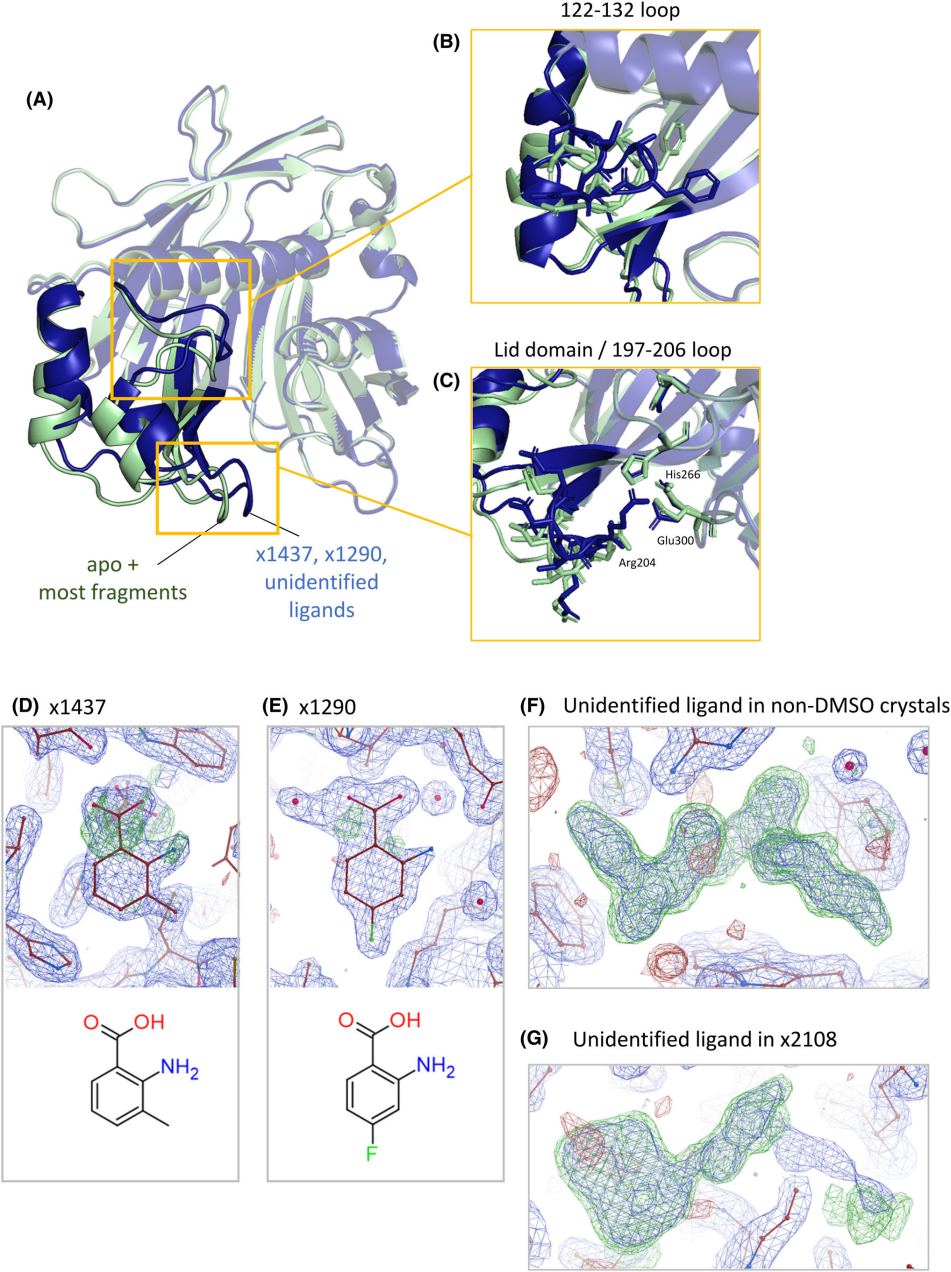
Some ligands drive large conformational changes within the N‐terminal half of FatA. (A) Overview of conformational changes induced from an overlay of x1163 and x1437 crystal structures. Two regions of interest are identified–122–132 loop and a lid domain. (B) Close‐up of 122–132 loop movements especially the position of Phe128. (C) Close‐up of the lid domain/197–206 loop movement towards the active site loop. These changes were observed in datasets containing: fragments (D) x1437 and (E) x1290. (F) Unidentified density in crystals without exposure to DMSO (x0045) obtained during crystallization before the fragment screen, and (G) unidentified density in chain B of x2108.

Upon binding of some ligands, large portions of the N‐terminal Hotdog domain of one chain, chain B, move closer to the C‐terminal half relative to the *apo* state. Specifically, two regions composed of residues 122–142 and residues 195–220 [Fig. 5(A)] move 2.3 Å and 3.3 Å, respectively. Both regions are proximal in space and are located at the distal end of the substrate/herbicide binding site. Concurrently, these areas displayed higher B‐factors compared to the rest of the structure, suggesting that they remained somewhat dynamic after movement (Fig. S6).

Specifically, we found four chemical entities which caused the same significant shifts within chain B – all bound at the herbicide binding site. First, the conformation was observed in crystals which have not been exposed to DMSO, such as x0045, in which the herbicide binding site is occupied by an unidentified ligand, likely to be an *E. coli* metabolite [Fig. 5(F)]. Secondly, the shift is observed within the dataset x2108, where the herbicide binding site of chain B is occupied by another unidentified density which does not resemble the expected fragment (captured at chain A), likely to be a fragment impurity such as a leftover reactant or product [Fig. 5(G)]. Finally, two fragments from datasets x1290 and x1437 which share a similar chemical motif and are both bound at the herbicide binding site both caused the same shift [Fig. 5(D),(E)].

Another dataset, x2025, was captured in an intermediate position between the majority of fragments and the four discussed above (Fig. S7). It does not resemble fragments from x1290 to x1437 and its loop 122–132 is in a different conformation, and thus it is closer to the rest of the fragments than the outliers.

**Figure 6:**
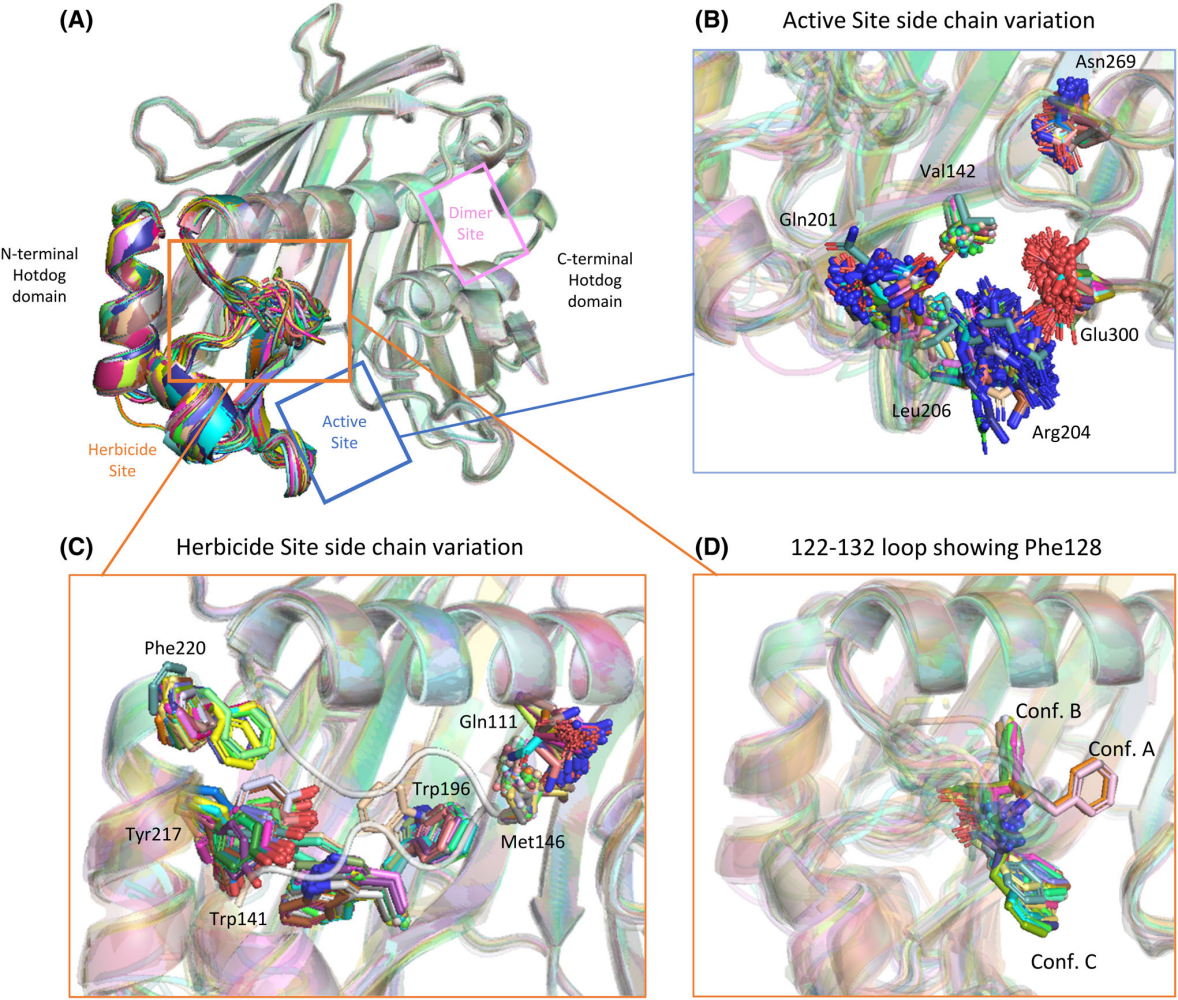
Higher conformational flexibility exhibited by the N‐terminal half of FatA than the C‐terminal half. (A) Overview of conformational flexibility from an overlay of all fragment bound structures. The herbicide and active sites located within the dynamic regions are highlighted in orange and blue respectively. Side‐chain movements inside the (B) active and (C) herbicide sites. Specific side chains which show particularly large conformational flexibility are shown and labelled. (D) Close‐up of the 122–132 loop flexibility as seen through three possible positions of Phe128.

Two regions of functional importance were identified to be contained within the mobile region. The 122–132 loop, which might form a gate to the substrate binding cavity via a bulky Phe128, and the 197–206 loop, which previously has been identified as a lid region, mediating interactions with ACP [Fig. 5(B),(C)]. In datasets where such movement occurs, the 122–132 loop of chain B (where the ligand density is the strongest) becomes structured and Phe128, located on this loop, assumes a particular conformation which we defined as conformation A [Fig. 6(B)]. The large conformational movements also caused the tip of the 122–132 loop and the lid domain to move closer to the active site loops which reduces the size of the substrate access channel.^21^

### 2.5 Substrate‐binding N‐terminal half of FatA displays much greater conformation variability than the catalytic C‐terminal half

When comparing the structures obtained during the fragment screen which did not cause the large conformational movements outlined above, we noticed that the N‐terminal Hotdog domain of FatA, which is responsible for substrate binding and specificity, displays a greater degree of conformational variability when compared to the rest of the protein [Fig. 6(A)].^19^ This affected the herbicide binding site and the active site, whereas the dimer site, despite being formed of residues from both A & B chains of FatA, showed little crystal‐to‐crystal variability.

#### 2.5.1 A loop adjacent to the herbicide binding site displays a great deal of conformational variation

The 122–132 loop which forms a part of the substrate binding site was observed in a multitude of different conformational states [Fig. 6(B)]. Specifically, the density of residues 124–129 was too poor to allow modelling in most datasets. However, upon binding of certain fragments, these residues became ordered and could be modelled. This stabilization of the loop was accompanied by a slight improvement in the overall resolution from an average of 2.03–1.83 Å (Fig. S8). Where loop 122–132 could be modelled, it undertook one of three conformations that we have annotated conformation A, B and C, based on positioning of Phe128 [Fig. 6(B)]. An analysis of the RMSD between the crystal structures corroborated these observations (Fig. S6). As mentioned above, conformation A was only observed together with large conformational changes. It is the only conformation of the three to deviate slightly from a preferred rotamer and thus carry an energetic penalty for the ligands bound here (Fig. S9). Another conformation, B, is predicted to clash with a modelled C18:1 and thus would cut off access to the substrate‐binding cavity, offering an additional mechanism of inhibition for the fragments and herbicides.

Side chains of residues which lie on this loop, such as Phe123, Ala129 and Thr130, were captured in a variety of conformations. The low density and subsequently high B‐factors reflect the relative mobility of these residues and the rest of the loop. Of those, Phe123 frequently interacts with fragments bound here.

#### 2.5.2 Residues involved in key interactions within the herbicide binding site displayed high conformational variability

Many of the residues located at the herbicide binding site adopted several conformations [Fig. 6(C)]. Tyr217, which makes the most interactions with fragments out of all residues and binds some herbicides, was observed in several conformations. However, the density was well‐resolved in most cases, suggesting that, unlike the residues of the 122–132 loop, Tyr217 adopts stable conformations. Likewise, Phe220 was observed in two different conformations, again with good density to support each. When fragments bound nearby, the side chain moved away ≈100° but still remained a preferred rotamer. Both of these residues are located on a *α*‐helix 3, which moves during the global movement.

Two tryptophan residues which line the substrate binding cavity, Trp141 and Trp196, were generally observed in the same position, but in rare instances their side chains were flipped 90°, although remaining a preferred rotamer [Fig. 6(C)]. Specifically, the fragments x1620 and x1747 which both contain a phenylmethoxy group stabilize the flipping of the Trp141 which brings the end of its ring within 4 Å of the fragments. x1620 also stabilized a flipped conformation of Trp196 (Fig. S4(F)).

Some of the variability resulted from poorly resolved density of side chains such as in the case of Gln111, Met146 and Thr174. The flexibility of these side chains may promote their interactions with a greater set of fragments. Two cysteines, Cys115 and Cys166 were often observed in two alternate conformations.

#### 2.5.3 The active site possesses lots of flexible side chains

High side‐chain flexibility within the active site was observed, both from residues on the N‐terminal loops, such as Arg204, Leu201, Leu206 and Val142, and from the C‐terminal half. Of the catalytic residues, Glu300 and Asn269 displayed particularly reduced densities indicative of movement [Fig. 6(D)]. Because of this ambiguity, they have been modelled in a variety of conformations, each with reduced certainty. Such conformational flexibility may be necessary for the accommodation of the large diversity of chemistry we observed to bind here; some bound fragments would clash with side‐chain conformations observed in other datasets.

### 2.6 Measurable inhibitory activity is already displayed by some fragments

**Figure 7:**
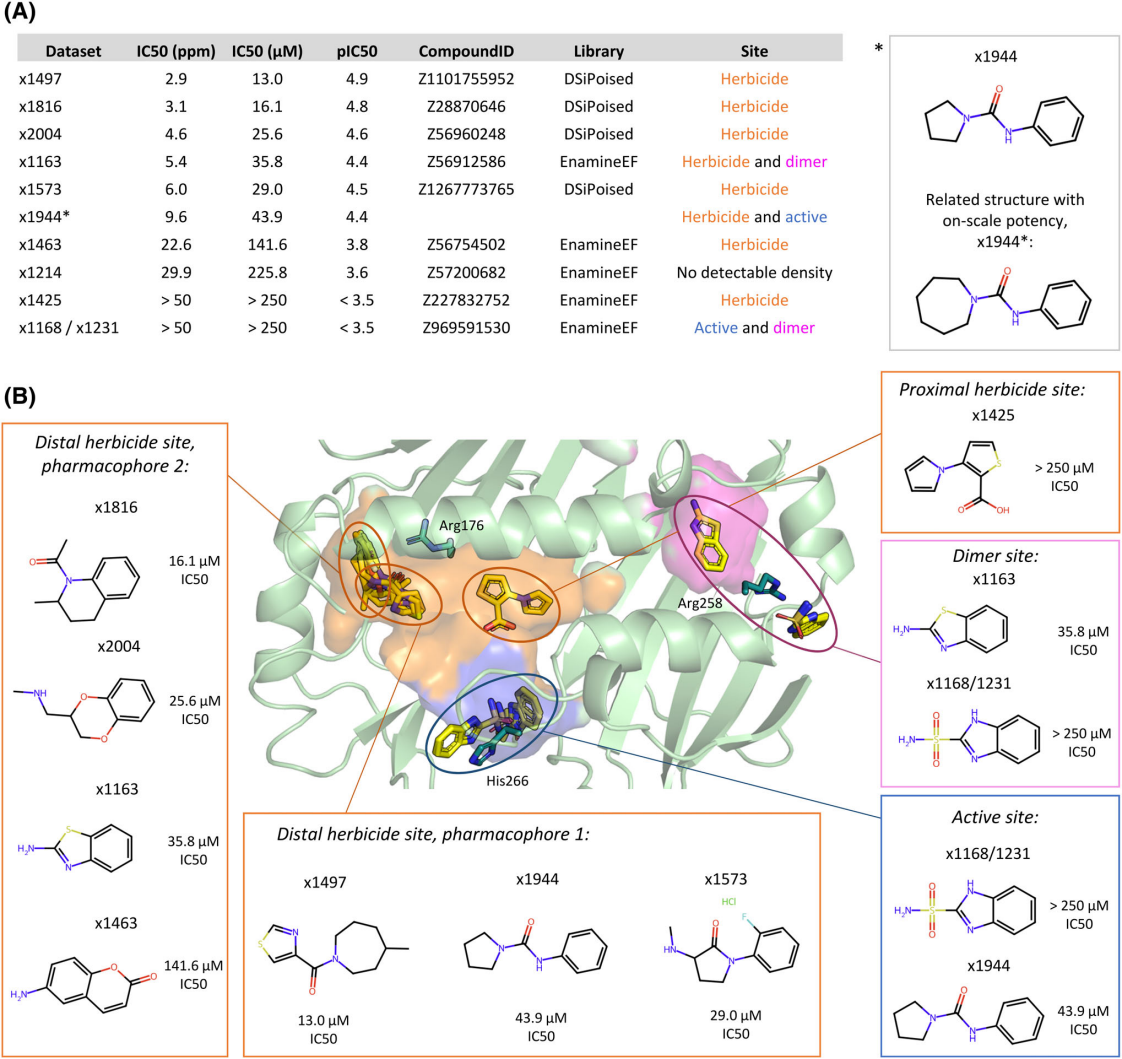
Ten fragments have on‐scale potency when tested in an enzymatic assay with FatA. (A) Summary table of the identity and inhibitory activity of the 10 fragments. (B) Binding poses of the nine fragments and their chemical structures. The surface has been produced in PyMOL using the cavities and pockets (culled) representation; the cavity detection radius and cutoff were both set to five solvent radii.

Sixty four fragments were available for testing in an *in vitro* enzyme assay. In this assay, the ACP cofactor was replaced by CoA and a thiol‐reactive fluorescent dye was used to quantify the enzyme's activity. Ten fragments were found to have on‐scale potency with the top compound achieving a 13.0 μm IC_50_ (Figs 7 and S10; raw data are available in Data S2). One of these fragments, x1214, produced a PanDDA event but did not result in convincing fragment density during refinement, leaving its binding site ambiguous. One fragment, x1944, did not demonstrate detectable inhibition, but an analogue where the pyrrolidine ring was substituted with an azepane to produce x1944*, had an IC_50_ of 43.9 μm [Fig. 7(A)]. Most of these fragments bound at the herbicide binding site and produced clear electron density.

x1168/x1231 bound at the active site and produced detectable activity, however, the IC_50_ value was too high to be determined accurately. It formed a *π*‐stacking interaction with catalytic His266 and a water bridge with Asn269 [Fig. S4(G)].

At the herbicide binding site, two main pharmacophores were observed [Fig. 7(B)]. Pharmacophore 1, shared by x1497, x1573 and x1944, resembled the current herbicides; they formed an H‐bond with Arg176 while also possessing hydrophobic entities on either side of the H‐bond acceptor to interact with the greasy cavity. Pharmacophore 2, shared by x1816, x2004, ×1163 and ×1463, resembled 1,8‐naphthyridine: these were bicyclic, aromatic compounds which bound closer to the deep end of the pocket. Although no interaction was common to all four, Val121, Phe123, Arg176 and Phe220 made interactions with three of the compounds. These were mainly hydrophobic, with occasional *π*‐stacking and *π*–cation interactions via the rings. The last fragment, ×1425, bound within the proximal end of the cavity and made both hydrophobic and H‐bonding interactions with Gln111 and Asn269 [Fig. S4(H)].

### 2.7 The fragment screen offers several diverse opportunities for elaboration

Because of the disjointed nature of the three binding sites, separate elaboration of fragments bound at each site is required. Fragment growing, merging and linking are the common strategies for fragment progression. As far as elaboration hypotheses, three principal means of inquiry have emerged: elaboration based on conformational changes, prioritization of compounds with measurable inhibition activity and/or maximization of the number of key interactions.

**Figure 8:**
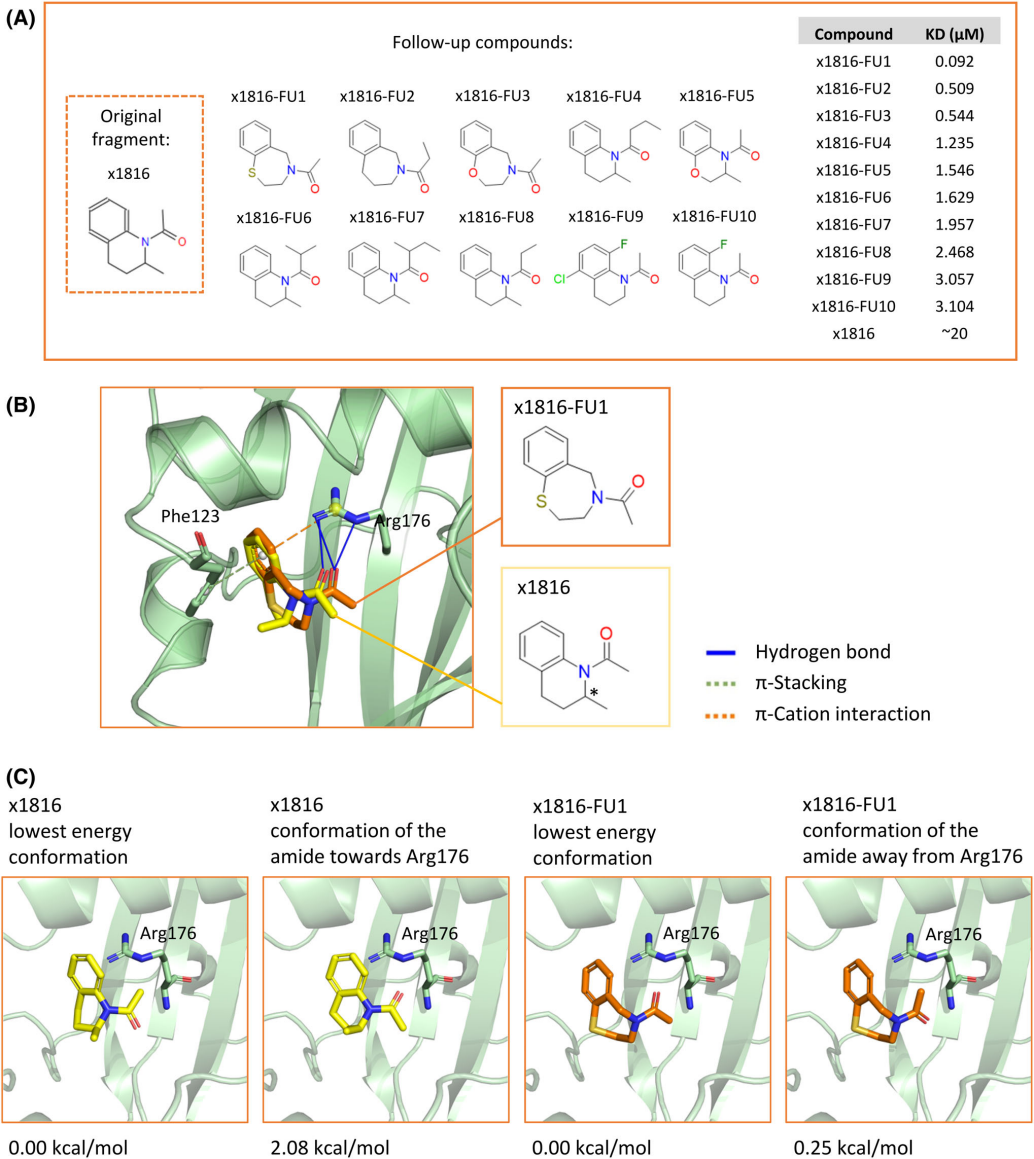
Expansion of a single fragment, x1816, elucidates the SAR: the increased ring size of x1816‐FU1 allows the amide greater opportunity to bind to Arg176 and relieves ligand strain. (A) Top 10 compounds with the best affinity from an exploratory campaign around fragment x1816. Chemical structures and *K*_D_ from Surface Plasmon Resonance (SPR) data are shown. (B) Overlay of the crystal structures of x1816 (shown in yellow) and x1816‐FU1 (shown in orange), with binding pose is preserved. The aromatic rings are ‘anchored’ by *π*‐stacking to Phe123 (shown as a dashed green line) and *π*–cation interaction with Arg176 (shown as dashed orange line). x1816 makes one H‐bond to Arg176 whereas x1816‐FU1 makes two (shown as solid blue lines). Chemical structures of the compounds are shown. The chiral centre of x1816 is marked by an asterisk. Figure was partially made with the Protein Ligand Interaction Profiler (PLIP). (C) Possible conformations of x1816 and x1816‐FU1, and the associated energies as deduced from quantum mechanical calculations with implicit solvent models (both hexane and water).

A limited elaboration campaign to explore close analogues of the active x1816 fragment was conducted and improvement in binding affinity was observed by Surface Plasmon Resonance (SPR). The analogues were used to explore the binding capabilities of the herbicide pocket as well as tolerance to heterocyclic ring size. Analogues of x1816 fragment were selected from the Enamine Database based on Tanimoto similarity and a substructure match; they were then further filtered based on docking results. The resulting 60 compounds were either purchased from Enamine REAL or synthesized in‐house. Of those, 27 had improved affinity when compared to the parent fragment which had a 17–20 μm *K*_D_. The top compound, x1816‐FU1 had a ~ 90 nm *K*_D_ constituting a ×220 improvement from a single design‐make‐test‐analyze cycle. Structures and affinities of the top 10 follow‐up compounds are shown in Fig. 8(A). Chemical structures of all x1816 analogues tested are available in Data S1. Binding data can be found in Data S2.

In order to investigate the origin of the enhanced binding affinity between x1816 and x1816‐FU1 we have solved the structure of FatA bound by x1816‐FU1 (PDB ID: 9S4H). We note that the binding pose between the two has been preserved [Fig. 8(B)]. The precise reason for the gain in affinity would require more extensive investigations outside the scope of this publication; beyond the elimination of a racemic stereocentre, we note several contributing factors. Because both ligands are ‘anchored’ in position by their aromatic ring *π*‐stacking to Phe123 and the *π*–cation interaction with Arg176, the increased ring size of the second ring allows the amide greater opportunity to bind to Arg176. When comparing our crystal structures, x1816 only forms one H‐bond to HH of Arg176 whereas x1816‐FU1 forms two with both HH and HE [Fig. 8(B)]. Moreover, quantum mechanical calculations with implicit solvent models (both hexane and water) indicate that the lowest energy conformation of x1816 is one where its amide carbonyl is facing away from Arg176, whereas the modelled conformation carries an ≈2 kcal mol^−1^ penalty (this alone could account for a 30‐fold change in potency) [Fig. 8(C)].^33^ By contrast, the lowest energy confirmation of x1816‐FU1 is one which can H‐bond with Arg176 [Fig. 8(C)]. Additionally, molecular dynamics simulations on equilibrated structures reveal that x1816‐FU1 forms on average slightly shorter H‐bonds to the Arg176 HE/HH protons (2.11 *versus* 2.17 Å).

**Figure 9:**
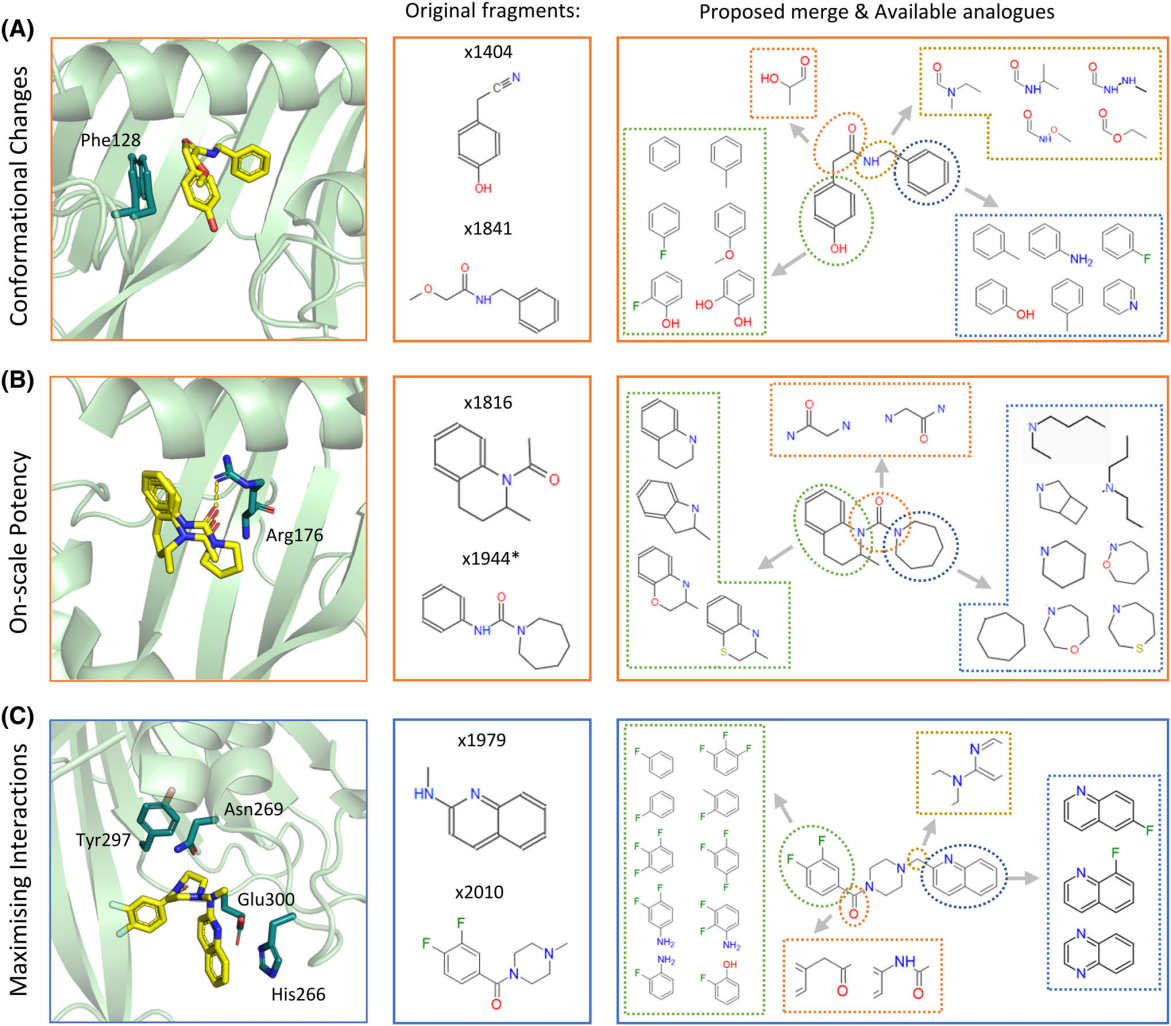
Fragments offer numerous merging opportunities (A). Examples of proposed fragment merges based on (B) conformational changes, (C) detectable inhibitory activity and (D) key interactions. Binding pose and structure of the original fragments, chemical structure of the proposed merge and closest purchasable compounds are shown.

Merging opportunities also have emerged which can explore the hypotheses outlined above. For example, to recapitulate a closed loop conformation, two fragments can be merged which both have caused the closed conformation of Phe128 on the 122–132 loop (conformation B), such as x1404 and x1841 [Fig. 9(A)]. Alternatively, to explore the large shifts within the N‐terminal domain, fragments x1437 and x1290 can be merged combinatorially.

When looking at compounds with measurable inhibition activity, most have bound at the distal end of the herbicide pocket and fall into one of two aforementioned pharmacophores (those that H‐bond to Arg176 and resemble current herbicides, and bicyclic compounds resembling 1,8‐naphthyridine). Compounds of each group are quite similar, but the combination of the two yields some promising structures such as ×1944 and ×1816 [Fig. 9(B)].

Lastly, when trying to maximize the interactions made by the follow‐up compound, we picked sets of overlapping fragments that interact with key residues of the site and together span the largest area. For example, the joining of the deep and shallow sites within the active site through a merge between ×1979 and ×2010 could maintain H‐bonds with Asn269, salt bridge to Glu300 and crucially, *π*‐stack with the catalytic His266, as well as expand to the hydrophobic network of Tyr297, Ile140 and Val142 [Fig. 9(C)]. Inside the herbicide site expansion of the distal end will lead to recapitulation of the interactions made by current herbicides, whereas the proximal end contains newly identified key interactors such as Gln111, Met146, Tyr297 and Asn269.

## 3 DISCUSSION AND CONCLUSIONS

Our crystallographic fragment screen has revealed the large conformational flexibility of FatA. This may be how FatA has adapted to receive large substrates and, in turn, creates an opportunity that may be exploited by inhibitors. The N‐terminal half of FatA which is responsible for substrate binding is much more dynamic than the catalytic C‐terminal half as evident from the large shifts observed in some datasets, the dynamic nature of the nearby loop 122–132 and the extensive side‐chain movement.^19^ What remains to be seen is if binding of the substrate forces these movements in an induced‐fit model or whether FatA samples various conformations which are then selected by its substrate or its ligands. Inhibitors bound at the herbicide site may not only sterically exclude the substrate, but also lock FatA in a closed conformation so that the FA cannot enter the substrate access channel at all. These observations will guide follow‐up compound design through prioritization of fragments which caused more radical movements and merging of fragments which have caused similar structuring of the mobile elements.

The herbicide binding site, named herein for its binding to all known FatA inhibiting herbicides, is both highly populated and effective. The site is located deep within the central cavity formed between the *β*‐sheet and the *α*‐helix of the N‐terminal side of FatA. It has evolved to receive FAs and is predictably highly hydrophobic. Fragments bound here traversed the long channel to make mostly hydrophobic interactions, although H‐bonds with Arg176 and Tyr217 also were frequently observed. Notably, a subset of fragments which bind here cause the large conformational changes and structuring of the 124–129 residues. Additionally, the majority of fragments with on‐scale potency against FatA bind at this site. The more enclosed nature of the herbicide binding site may contribute to the greater efficiency of inhibitors bound here [Fig. 7(B)].

The fragment screen has revealed orthogonal opportunities for novel inhibitor development. Some of our fragments bind at the herbicide binding site, but explore novel interactions and space when compared to current herbicides. Their further development would alleviate mutational pressure from the residues currently key to herbicidal interactions. We also have found two other sites which show promise, including one fragment (x1168/x1231) which binds at both and has shown detectable activity. The active site is closer to the surface and thus more accessible than the herbicide site, and it is likely that bigger ligands will be required to produce a greater inhibitory effect. However, we have found fragments that interact with the active site residues themselves and thus show great promise as starting points. The dimer site also is interesting because dimerization has been shown to be necessary for FAT activity.^21^ Merged compounds could be designed to pry the two chains apart or affect catalysis allosterically, although at the risk of stabilizing the dimer instead. However, the entropic cost of binding is likely to be lower here becasue this site is located mainly in the C‐terminal half which is much less dynamic.

The large number of bound fragments has readily lent itself to growing and merging opportunities, even when inspected by eye. Merging especially has been shown to be an effective way of quickly accessing on‐scale kinetics and potency.^34^, ^35^, ^36^, ^37^ SAR exploration may then be achieved by purchasing the merged compounds and their analogues. Moreover, a small elaboration campaign using close analogues of an active ×1816 fragment has already yielded an improvement in affinity from ~20 μm to ~90 nm.

Inhibition of both FatA and FatB is necessary to see lethal effect and must be considered when developing novel inhibitors. *A. thaliana* FatA and FatB have a 47% sequence identity and a 64% similarity (Fig. S11). The two also possess the same catalytic residues. As such, inhibitors targeting the active site are likely to inhibit both enzymes. The variation between the two families stems from their N‐terminal domains which dictate substrate specificity. Because this is where the herbicide binding site is located, it is possible for the inhibitors to bind here differentially. However, many key residues involved in known herbicide binding modes, such as Arg176, Phe220, Trp141 and Trp196, are conserved. Some residues such as Val121, Phe123 and Tyr217 do differ between FatA and FatB, yet remain hydrophobic. For example, Phe123 is a leucine in FatB. It is thought that known herbicides must target both families as inhibition of both is necessary for the lethality displayed by the herbicides.^2^, ^17^, ^18^ However currently, *in vitro* testing is done on FatA only.^2^, ^4^, ^7^, ^8^ Failure of inhibitors with *in vitro* activity against FatA in *in vivo* studies could be attributed to lack of FatB inhibition. As such, moving forward it would be beneficial to test prospective leads against FatB.

Detailed understanding of structural biology and interaction capabilities of FatA, complemented with experience from pharmaceutical drug discovery, opens up new opportunities for compound development. It is crucial to pre‐empt and avoid mechanisms of resistance; this is especially valuable as FatA herbicides show promise as a route to control weeds resistant to existing herbicides.^6^ What is specifically relevant here are the related concepts of *substrate envelope* and mutational fitness costs, developed in antiviral discovery: these approaches entail identifying the specific interaction opportunities in the targeted binding site, that the enzyme must absolutely rely on for its activity; and then exploiting these exclusively for improving potency while developing the chemical series.^38^, ^39^ Such strategies not only require protein‐ligand structures to be reliably generated, which our protocols here provide, but also the very thorough understanding of the conformational and interaction landscape of the sites yielded by this fragment screen.

In summary, conducting a crystallographic fragment screen enabled us to find many new starting points for future inhibitor development. Concurrently, obtaining more than a hundred crystal structures of FatA revealed conformational flexibility of its substrate‐binding N‐terminal half. This illuminates a possible mechanism of substrate recognition and inhibition. These insights can accelerate rational follow‐up compound design, thus demonstrating the utility of structural fragment‐based approaches as tools in herbicide discovery and development.

## 4 MATERIALS AND METHODS

### 4.1 Protein expression and purification

A pET‐24a(+) plasmid containing codon optimized *A. thaliana* FatA sequence was supplied by Syngenta. The N‐terminal chloroplast transit peptide (residues 1–74) had been removed and a C‐terminal His_6_ tag added. See Table S1 in  Data S1 for the gene and protein sequence.

In order to express FatA, the plasmid was transformed into BL21(DE3) *E. coli* and grown overnight at 37 °C on lysogeny broth (LB) agar containing kanamycin (50 μg mL^−1^). Several transformed colonies were inoculated into super optimal broth with catabolite repression (SOC) media containing kanamycin (50 μg mL^−1^) and grown overnight at 37 °C. Ten millilitres of starter culture were used to inoculate 1 L of terrific broth containing 10 mL glycerol and 50 μg mL^−1^ kanamycin, and incubated at 37 °C, 200 rpm until an optical density of 1 was reached. Temperature was reduced to 18 °C and after 1 h, 0.5 mm isopropyl *β*‐d‐1‐thiogalactopyranoside (IPTG) was used to induce protein expression. Cells were grown overnight at 18 °C, 200 rpm before being harvested by centrifugation and frozen at −80 °C.

The cell pellet was resuspended in 25 mm HEPES pH 7.5, 500 mm NaCl, 0.5 mm tris(2‐carboxyethyl)phosphine (TCEP), 25 mm imidazole and 0.03 μg mL^−1^ benzonase. Cells were disrupted by sonication and the lysate was clarified by centrifugation at 30 000 × *g*. Supernatant was loaded onto a column packed with Ni Sepharose 6 Fast Flow resin (17 531 802; Cytiva, Marlborough, MA, USA) equilibrated in 25 mm HEPES pH 7.5, 500 mm NaCl, 0.5 mm TCEP, 25 mm imidazole and 10% glycerol. After washing, the protein was eluted with 25 mm HEPES pH 7.5, 500 mm NaCl, 0.5 mm TCEP, 500 mm imidazole and 10% glycerol. The protein was concentrated using a 10‐kDa molecular weight cut‐off centrifugal concentrator (VS2002; Sartorius AG, Goettingen, Germany), and applied to an SRT‐10 SEC‐300 column (225 300–21 230; Sepax Technologies, Inc., Newark, DE, USA) equilibrated in 25 mm HEPES pH 7.5, 150 mm NaCl and 0.5 mm TCEP. The protein was concentrated to 21 mg mL^−1^, flash‐frozen in liquid nitrogen and stored at −80 °C.

### 4.2 Crystallization and structure determination

Crystals were obtained through the sitting drop vapour diffusion method using crystallization conditions previously identified by Syngenta. Crystals were grown at 20 °C in SWISSCI three‐well plates (SWISSCI, 3W96T‐UVP) with 30 μL reservoir solution containing 0.1 m MES pH 6.85 and 1.6 m ammonium sulfate. Crystallization drops consisted of 200 nL protein (8.3 mg mL^−1^), 100 nL reservoir solution and 20 nL seeding stock. Crystals could be harvested after 1 day and did not require additional cryoprotectants. Seeding stock was used to improve the consistency of nucleation. To make the seeding stock, several crystals were crushed using a melted tip of a glass Pasteur pipette, transferred into 50 μL reservoir solution, crushed by vortexing ≈10 times for 10 s with a seed bead (Fisher Scientific, 12 398 637), incubating briefly on ice in between, and used at 1:10 dilution.

Data was collected on beamlines I03 and I04 at Diamond Light Source and processed using Diamond's fully automated processing pipelines which include Xia2, DIALS, autoPROC and STARANISO with the default settings.^40^, ^41^, ^42^, ^43^ Further processing was done in ccp4i2.^44^ The dataset was phased with a FatA structure provided by Syngenta using phaser ^45^ and refined with refmac5.^46^ Model building was done in coot.^47^ The structures of FatA in the *apo* state and bound to an unidentified ligand have been deposited in the PDB under the codes 9HRR and 9HRQ, respectively.

### 4.3 Modelling of FatA, ACP and oleic acid

FatA crystal structure from ×1747 was chosen because it has the most open 122–132 loop. Because the loop in chain A was unstructured, chain B was copied into its place to make a symmetric molecule. Position of ACP was predicted in complex with an *apo* FatA dimer using ColabFold and then superimposed onto ×1747 in PyMOL.^48^, ^49^ The 2FatA:2ACP model was then minimized using FastRelax in pyrosetta.^50^ Position of the substrate was modelled based on 4KEH, a cross‐linked structure between fatty synthase dehydratase (FabA), the *E. coli* ACP (AcpP) and sulfonyl‐3‐alkynyl crosslinking probe (1R3).^31^ The ligand was placed in a three‐step process: (i) 4KEH and 2FatA:2ACP model were aligned in PyMOL and the ligand 1R3 was copied over from 4KEH; (ii) the 1R3 ligand was modified to be two linked moieties, phosphopantetheine (PNS) and a second moiety, either a transition state compound (custom structure) or oleoic acid (OLA); and (iii) the structure was then minimized again. The model has been deposited in GitHub (https://github.com/matteoferla/A-thaliana-FatA-modelling) and Zenodo (https://doi.org/10.5281/zenodo.14413325).

### 4.4 Crystallographic fragment screening, data processing and analysis

Two fragment libraries were screened at XChem,^25^ Diamond Light Source: Enamine's essential fragment library (320 fragments) and Diamond‐SGC‐iNEXT Poised (DSipoised) library including the EUbOpen extension (781 fragments). *Apo* crystals were obtained as described above. Two crystallization conditions were tested in a 100‐fragment pre‐screen: 0.1 m MES pH 6.85, 1.6 m ammonium sulfate and 0.1 m sodium cacodylate pH 6.85, 1.6 m ammonium sulfate. All fragments were in DMSO at 500 mm. Fifteen nanolitres of fragment stocks were transferred into the 300 nL crystallization drops using an ECHO liquid handler (Beckmann Coulter, Brea, CA, USA), equating to a final concentration of 23.8 mm and DMSO concentration of 5%. After incubating at 20 °C for 2 h the crystals were mounted using the Crystal Shifter (Oxford Lab Technology, London, UK) and flash‐cooled in liquid N_2_.

Data was collected on beamline I04‐1 at Diamond Light Source and processed using Diamond's automated processing pipelines as described above. Further processing was done in xchemexplorer which generates electron density maps with DIMPLE, generates ligand restraints using AceDRG (CCP4) or Grade (GlobalPhasing), creates an *apo* ground state model and uses pandda to find ligand‐binding events within the bound state models.^51^, ^52^, ^53^, ^54^ Ligand hits were built using pandda‐generated event maps, successive rounds of refinement with refmac and model building with coot.^46^, ^47^ Coordinates, structure factors and PanDDA event maps were deposited in the PDB under group deposition ID G_1002328.

Molecular interactions were analyzed using the Hit Interaction Profiling for Procurement Optimisation (HIPPO) python package, Protein‐Ligand Interaction Profiler (PLIP) web service, Schrödinger's maestro software suite and cross‐validated using PyMOL.^49^, ^55^, ^56^, ^57^

B‐factor and RMSD analysis was performed using PyMOL and knime.^49^, ^58^

### 4.5 FatA enzymatic assay

Compounds were tested for *in vitro* inhibition of *A. thaliana* FatA using an assay for detection of the CoA product released following hydrolysis of oleoyl‐coenzyme A (OCA) used as a surrogate substrate. Standards and test compounds were solubilized in DMSO and applied in 1 μL to the plate well. The assay was carried out as follows: a reaction mixture consisting of 12 nm FatA in assay buffer [50 mm Tris HCl pH 8.0, 150 mm NaCl, 12 nM *β*‐casein (C6905, Sigma‐Aldrich, Waltham, MA, USA)] was combined with test compounds in black 384‐well microtitre plates (Fluotrac 200, 781 076; Greiner, Kremsmünster, Austria) in a final reaction volume of 70 μL. Plates were incubated for 15 min at room temperature (RT) with mixing (500 rpm). The plate was read (excitation 390 nm, emission 470 nm) using an Infinite plate reader (Tecan, Männedorf, Switzerland) to check for autofluorescent test compounds. The enzyme reaction was then initiated by the addition of OCA (Sigma‐Aldrich O1012) to 10 μm final assay concentration (added as 10 μL of 80 μm stock solution in assay buffer) and incubated for a further 30 min at RT with mixing at 500 rpm. The reaction was terminated by addition of CPM solution [7‐diethylamino‐3‐(4‐maleimidophenyl)‐4‐methylcoumarin; Sigma‐Aldrich 96669)] at 20 μm final assay concentration [added as 10 μL of 180 μK stock solution in detection buffer (50 mm Tris HCl pH 8.0, 150 mm NaCl, 50% ethanol)]. Following 10 min incubation in the dark at RT, the adduct generated by reaction of CPM with CoA was measured by recording the fluorescence intensity as above. Test compounds were assayed in an 8 rate dose response with a top rate of 25 ppm using 1 in 5 dilutions. The standard, CSCA651647, was tested in an 8 rate dose response starting at 1 ppm using 1 in 5 dilutions. Untreated, maximum, controls used 1 μL of DMSO and minimum controls had a final assay concentration of 1 ppm CSCA651647.

Data was analyzed by nonlinear regression using prism (v10.1.2 for Windows; GraphPad Software, Boston, MA, USA, www.graphpad.com). The activity data were normalized using the fluorescence intensity from the standard (minimum control) and untreated (maximum control) samples. A curve was fitted using nonlinear regression [log(inhibitor) *versus* response‐variable slope (four parameters)], which enabled extraction of the IC_50_ values.

### 4.6 Surface plasmon resonance

FatA was immobilized on an NTA sensor chip (GE Healthcare, Chicago, IL, USA) using a Biacore 8K, captured via His‐tag and simultaneous amine coupling on EDC/NHS‐activated carboxyl groups (Immobilization buffer: 25 mm HEPES pH 7.4, 150 mm NaCl, 1 mm TCEP, 0.05% Tween 20). Equilibrium binding experiments were performed at 20 °C using a Biacore 8K instrument (GE Healthcare) with 25 mm HEPES pH 7.4, 150 mm NaCl, 1 mm TCEP, 0.05% Tween 20 and 2% DMSO as running buffer for compound testing. Compounds were injected over the chip in two‐fold dilution series either in multi‐cycle mode (60s/180s association/dissociation time) or single‐cycle kinetic mode (160 s/1800s association/dissociation time). Indicative *K*_D_ values were obtained by steady‐state affinity fitting to a 1:1 binding site model.

Compounds were drawn using chemsketch.^59^

## AUTHOR CONTRIBUTIONS

E.K. protein production, crystallography, XChem fragment screening, data analysis, wrote the first draft of the manuscript. F.v.D., N.P.M., M.G.M., K.S.E, L.O.V. supervised the research, read and edited the manuscript. J.C.A., X.N., C.W.E.T., R.C., L.K. read and edited the manuscript. M.G.M initial crystallography. N.P.M. and M.G.M. ×1816 follow‐up SAR exploration. M.F. protein production support. X.N., L.K. crystallographic support. X.N. PDB deposition support. M.P.F. created the substrate‐bound model of the biological assembly. C.W.E.T., D.F., J.C.A. XChem support. M.P.F. and L.O.V. chemical proofreading. P.H.H. enzyme assay. M.W. data analysis of the interactions. R.C. native mass spectrometry.

## FUNDING INFORMATION

E.K. would like to thank the EPSRC ICASE and Syngenta Studentship for support of this work. K.S.E thanks Alzheimer's Research UK. M.P.F. is supported by the RosetreesTrust (M940). M.W. is supported by the European Research Executive Agency (grant agreement ID: 101094131). L.O.V. thanks Alzheimer's Research UK for support (ARUK‐2021DDIOX).

## CONFLICT OF INTEREST

Authors declare no competing interests.

## Supporting information


**Data S1.** Supporting Information.

**Data S2.** Supporting Information.

## Data Availability

The crystallographic coordinates and structure factors of FatA in the *apo* state and bound to an unidentified ligand have been deposited in the PDB under the codes 9HRR and 9HRQ respectively. The resulting structures from the XChem fragment screen have been deposited in the PDB under the group deposition ID G_1002328. Crystal structures of FatA bound to cinmethylin, oxaziclomefone and methiozolin have been deposited in the PDB with the codes 9GRR, 9GS1 and 9HMT respectively. The model of the biological assembly of FatA bound to C18:1‐ACP has been deposited in GitHub (https://github.com/matteoferla/A-thaliana-FatA-modelling) and Zenodo (https://doi.org/10.5281/zenodo.14413325). The authors declare that all other data supporting the findings of this study are available within the paper and its supplementary information files.
