# Supplementary material for: Crystal structure of fatty acid thioesterase A bound by 129 fragments provides diverse development opportunities
Source: Pest Manag Sci. 2025 Sep 12;82(1):151–68. doi: 10.1002/ps.70199 (PMC12713704; doi:10.1002/ps.70199)

## Supplementary Material 1

**Supplementary Table 1**. Gene and protein sequence of FatA used.

| **Gene Sequence** |
| --- |
| atgggtagcctgaccgaggatggcctgagctacaaagagaagttcgtggtgcgcagctacgaagtgggcagtaataaaaccgccaccgtggagaccatcgcaaatctgctgcaggaagttggctgcaaccatgcacagagcgtgggttttagcaccgacggtttcgccacaacaacaaccatgcgcaagctgcatctgatctgggtgaccgcccgcatgcatatcgagatctacaagtacccggcctggggtgacgtggttgaaatcgaaacctggtgccagagcgaaagtcgtattggtacccgccgtgactggattctgaaggatagcgtgaccggcgaagttaccggccgtgccaccagcaagtgggtgatgatgaaccaggatacccgccgcctgcagaaagtgagcgatgacgtgcgcgatgagtatctggtgttttgcccgcaagagccgcgcctggcatttccggaggagaacaatcgcagcctgaaaaagatcccgaagctggaagacccggcccagtatagtatgattggcctgaaaccgcgccgcgcagatctggatatgaatcagcatgttaataatgtgacctatattggctgggtgctggaaagtatcccgcaggagattgtggacacccacgaactgcaggttatcaccctggactatcgccgtgaatgccagcaggacgatgtggtggatagcctgacaaccaccaccagcgaaattggtggcaccaatggcagcgcaaccagcggcacccagggtcataatgacagccagttcctgcatctgctgcgtctgagcggcgatggccaggaaattaatcgcggcaccaccctgtggcgcaaaaaaccgagtagccatcatcatcaccaccactaa |
| **Protein Sequence** |
| MGSLTEDGLSYKEKFVVRSYEVGSNKTATVETIANLLQEVGCNHAQSVGFSTDGFATTTTMRKLHLIWVTARMHIEIYKYPAWGDVVEIETWCQSEGRIGTRRDWILKDSVTGEVTGRATSKWVMMNQDTRRLQKVSDDVRDEYLVFCPQEPRLAFPEENNRSLKKIPKLEDPAQYSMIGLKPRRADLDMNQHVNNVTYIGWVLESIPQEIVDTHELQVITLDYRRECQQDDVVDSLTTTTSEIGGTNGSATSGTQGHNDSQFLHLLRLSGDGQEINRGTTLWRKKPSSHHHHHH |

**Supplementary Note 1.** Chemical structures of the fragment hits:


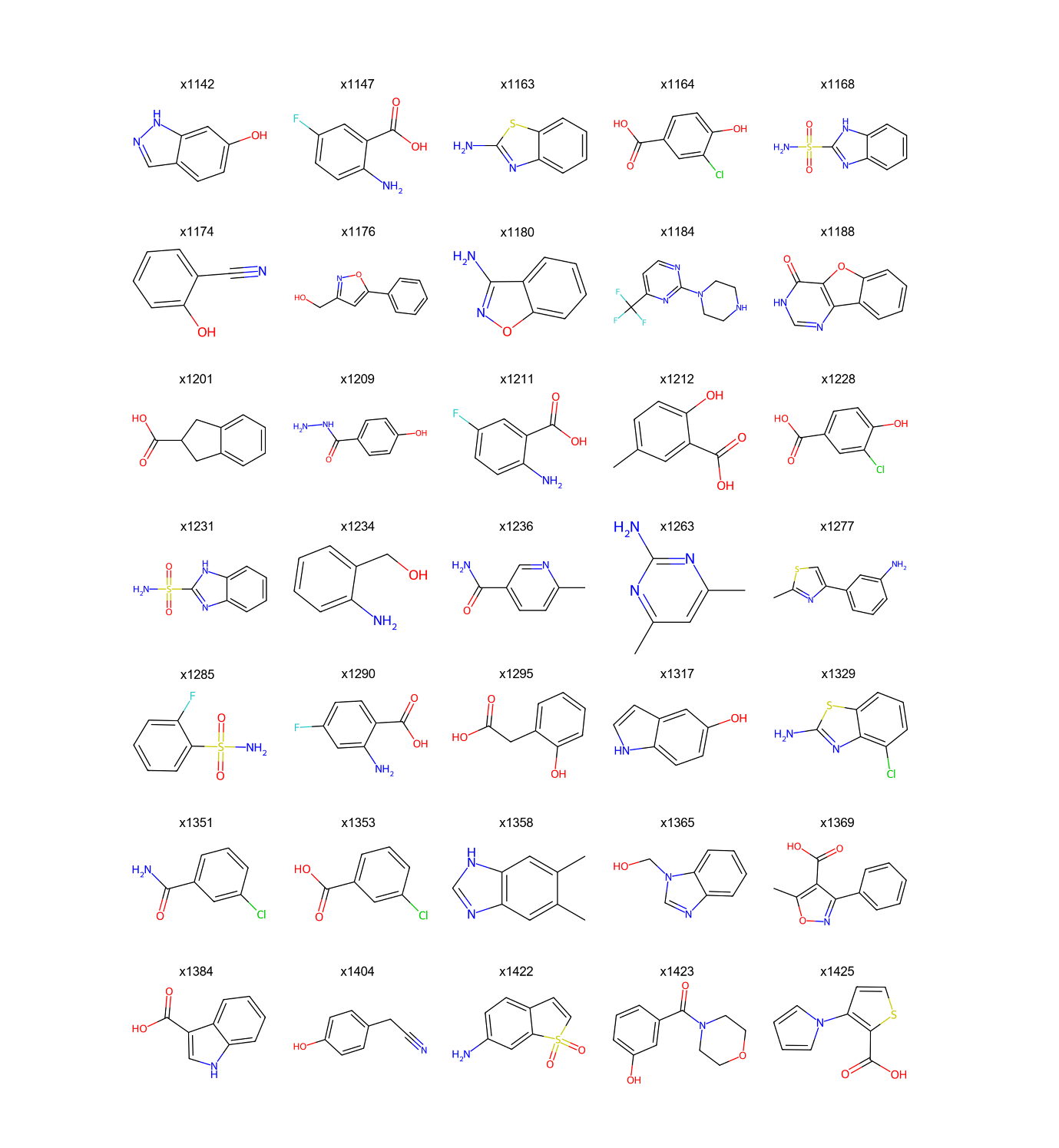


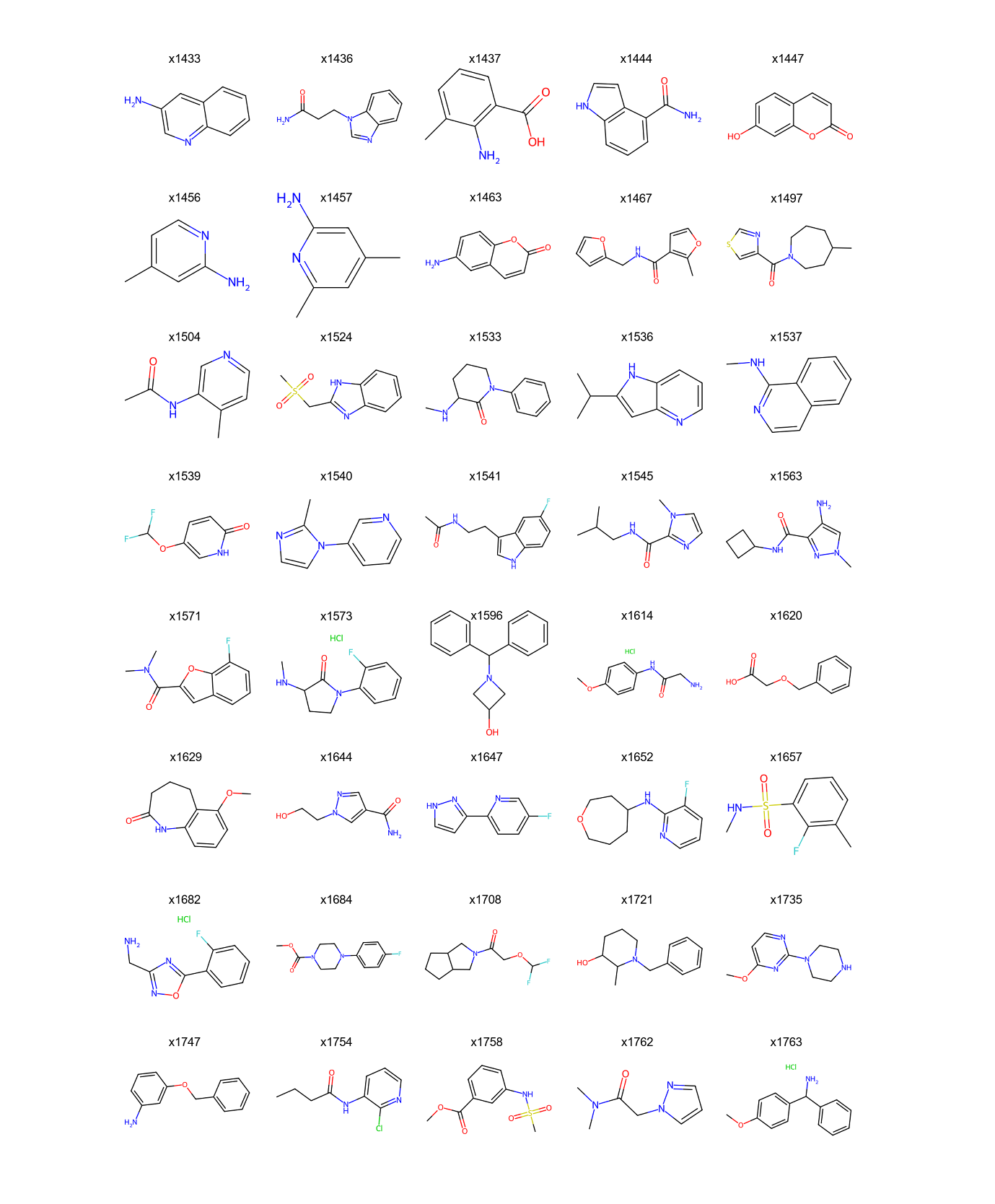


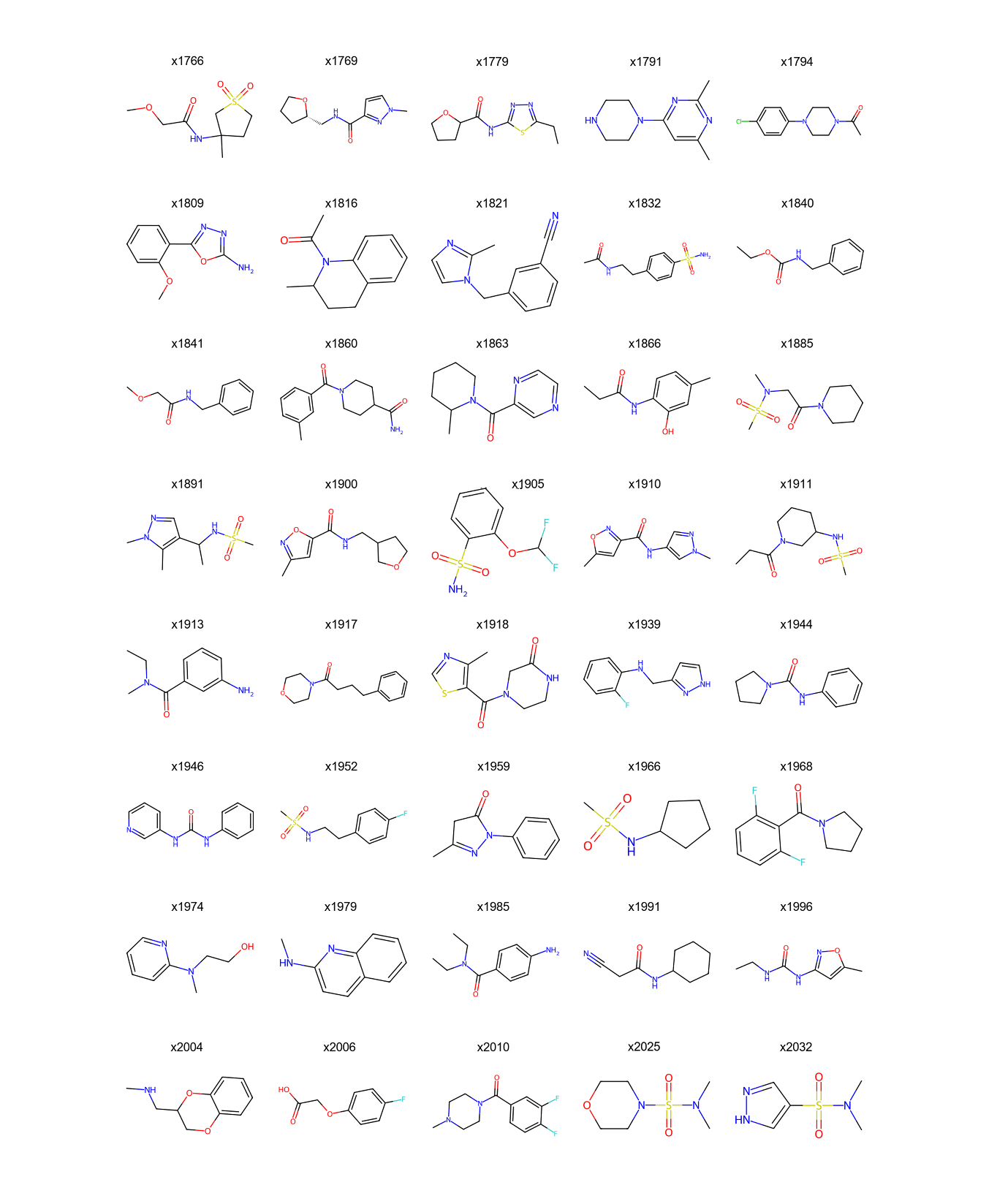


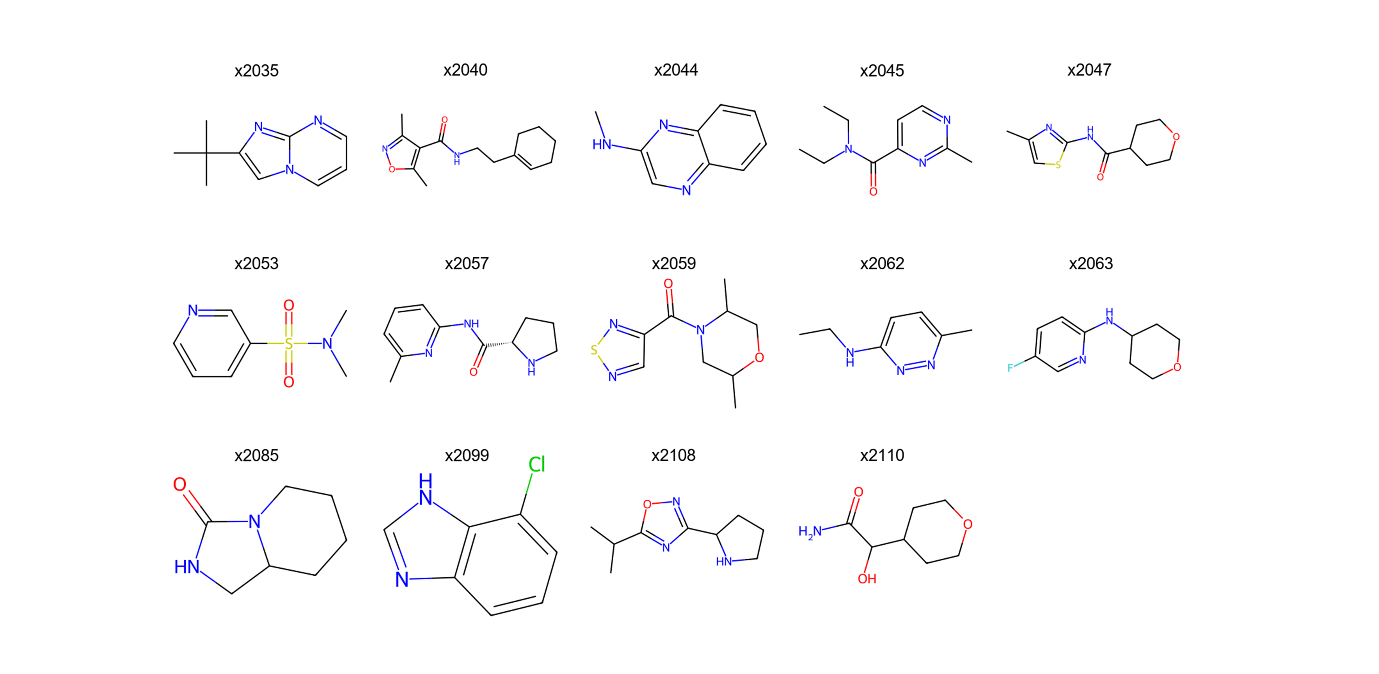


**Supplementary Note 2.** Chemical structures of the 60 tested analogues of x1816:


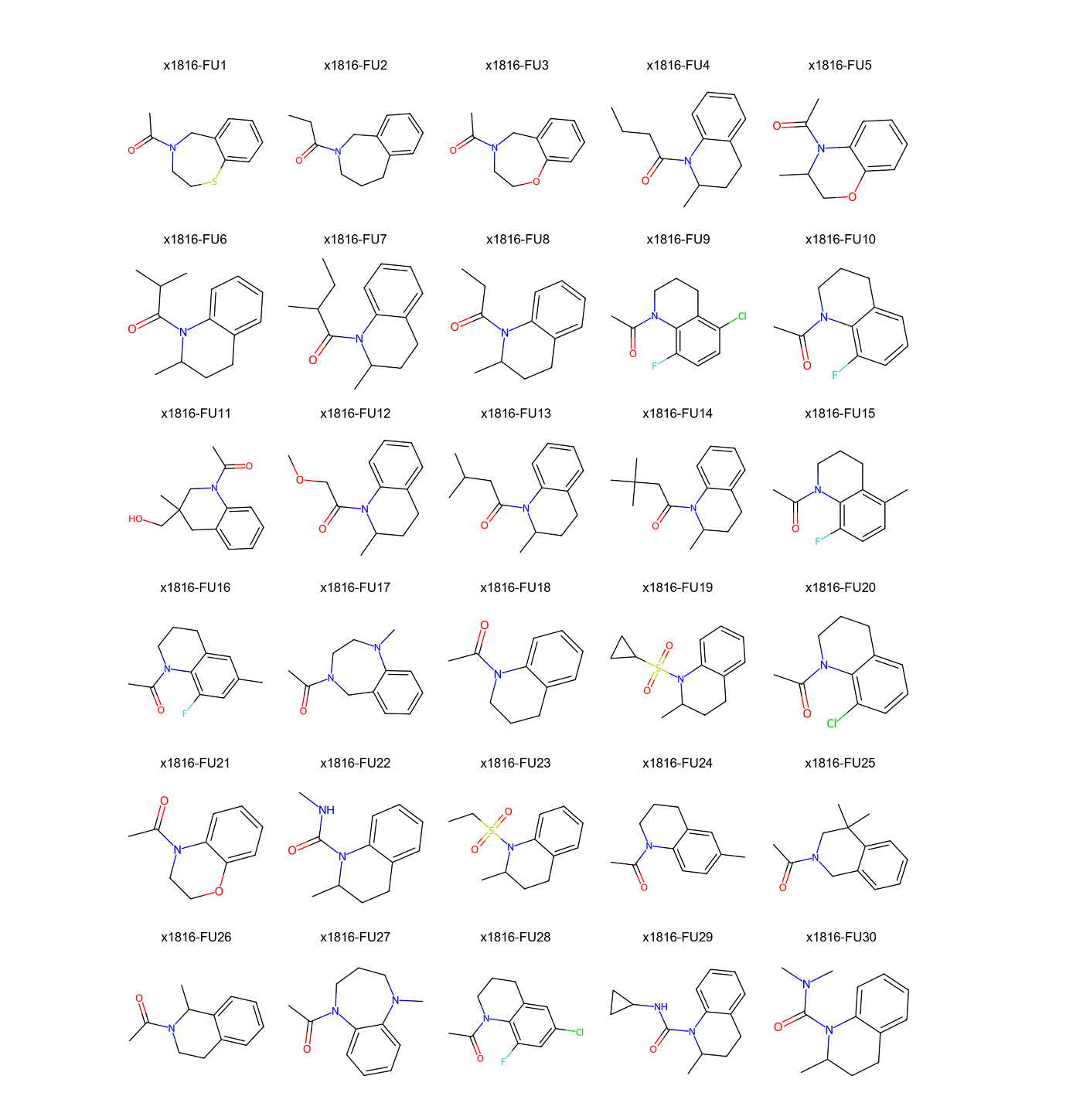


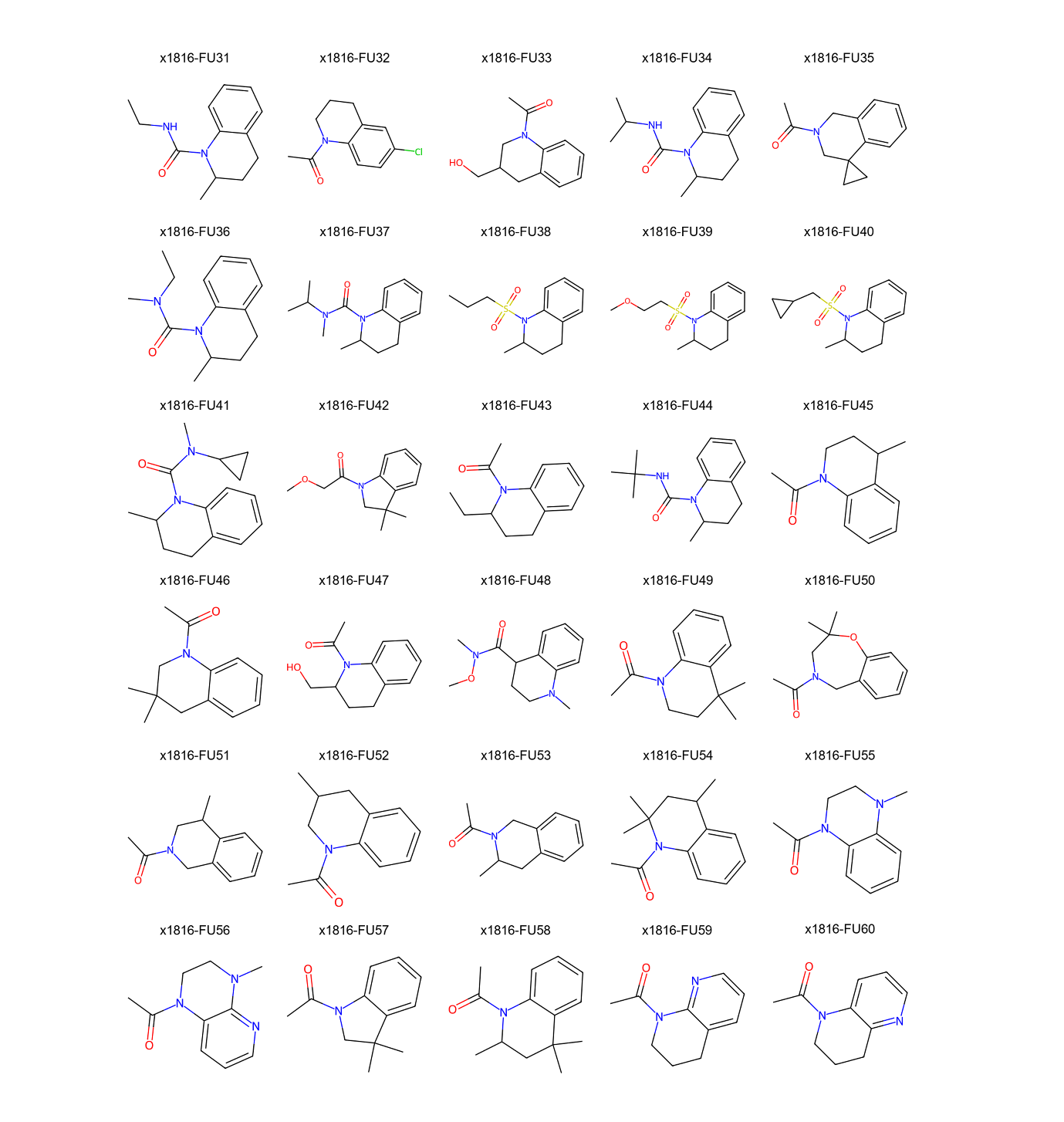


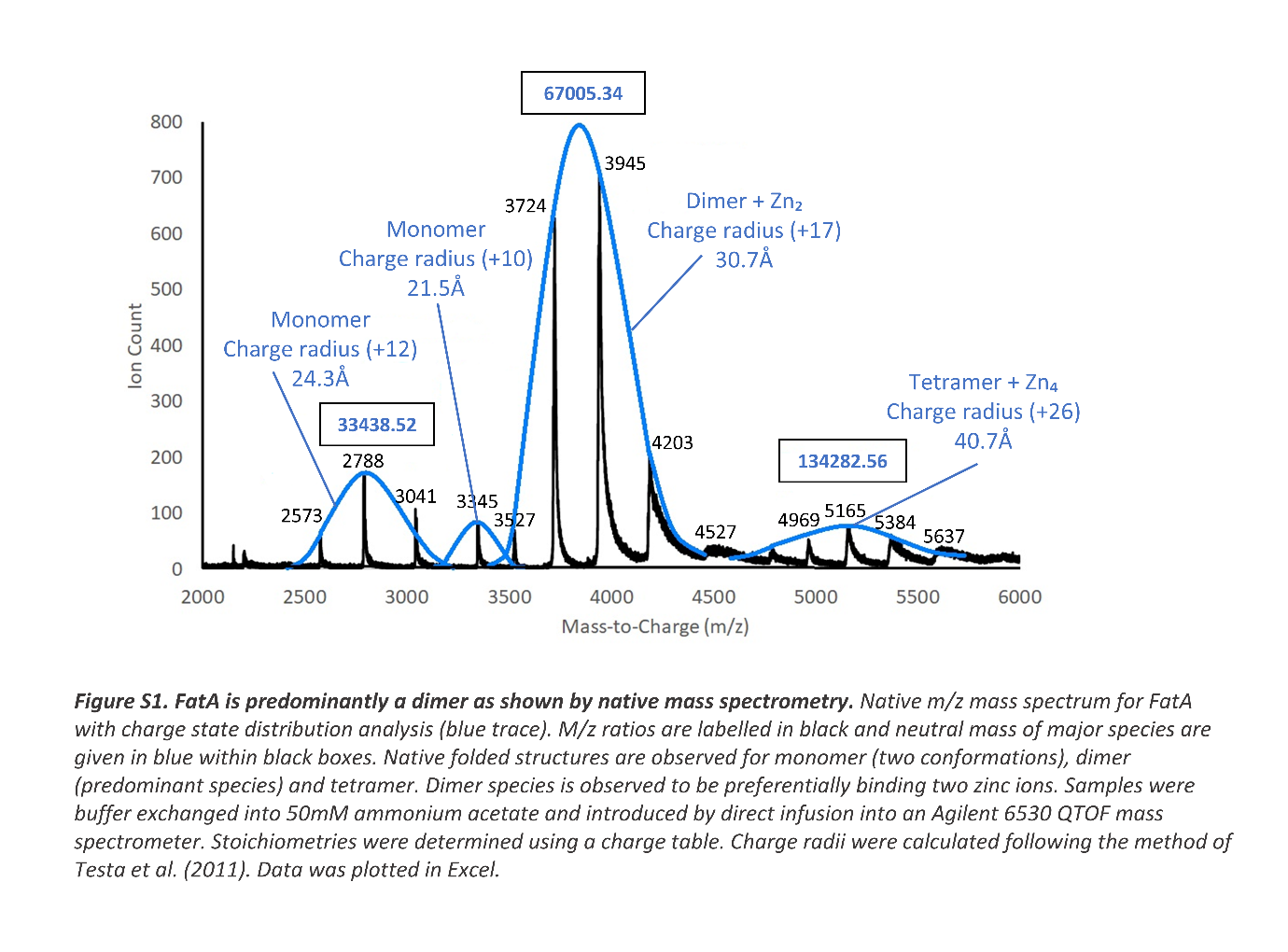


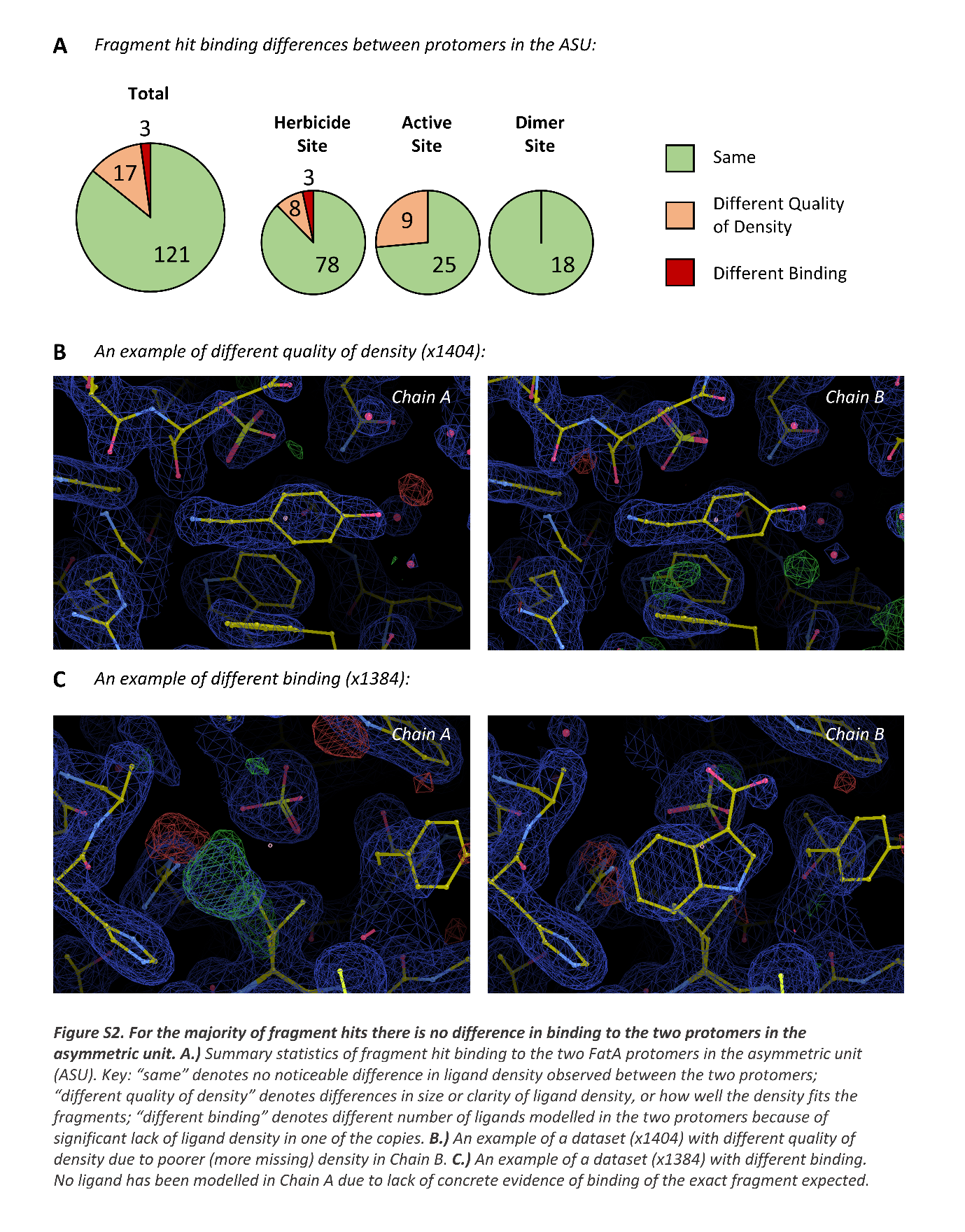


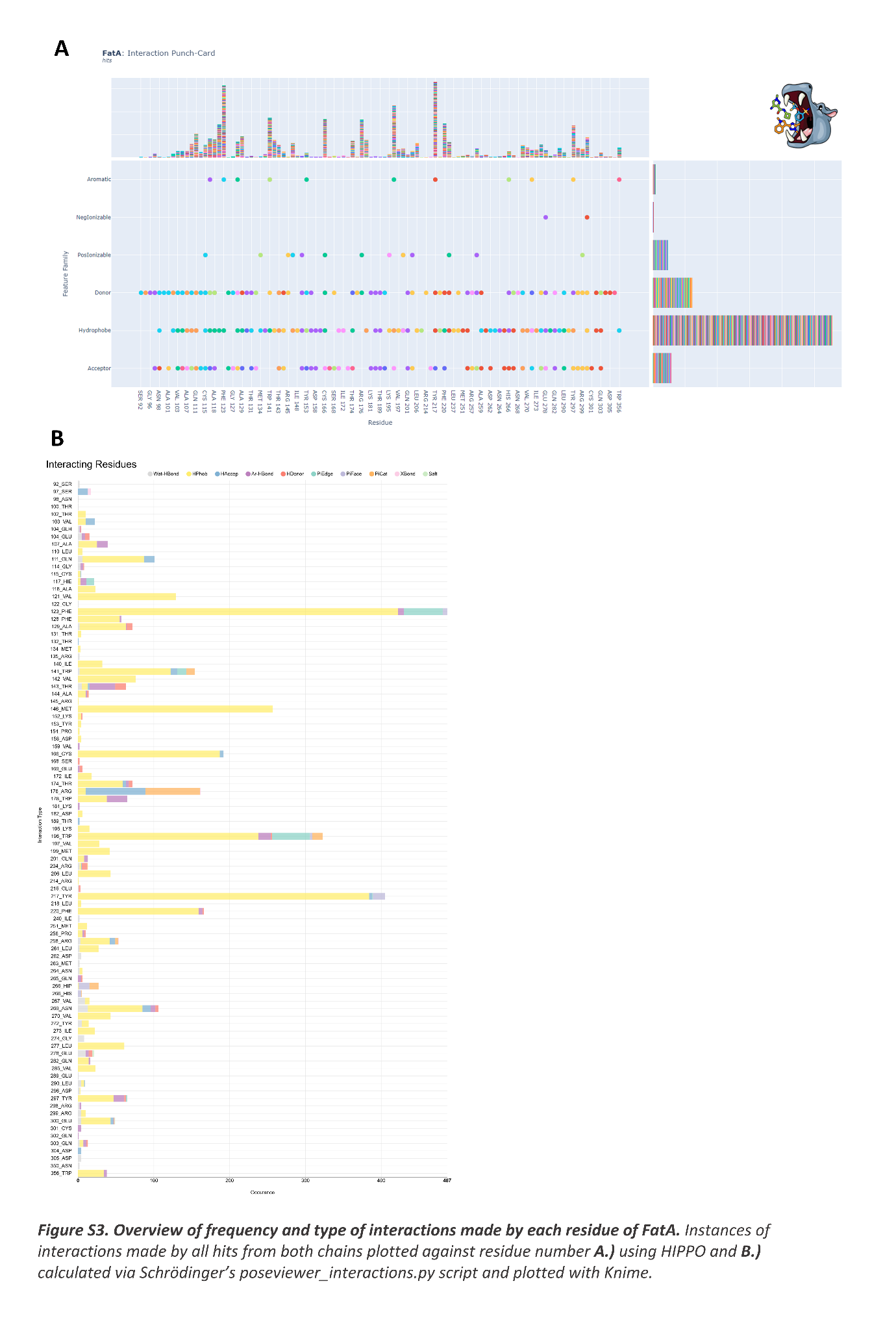


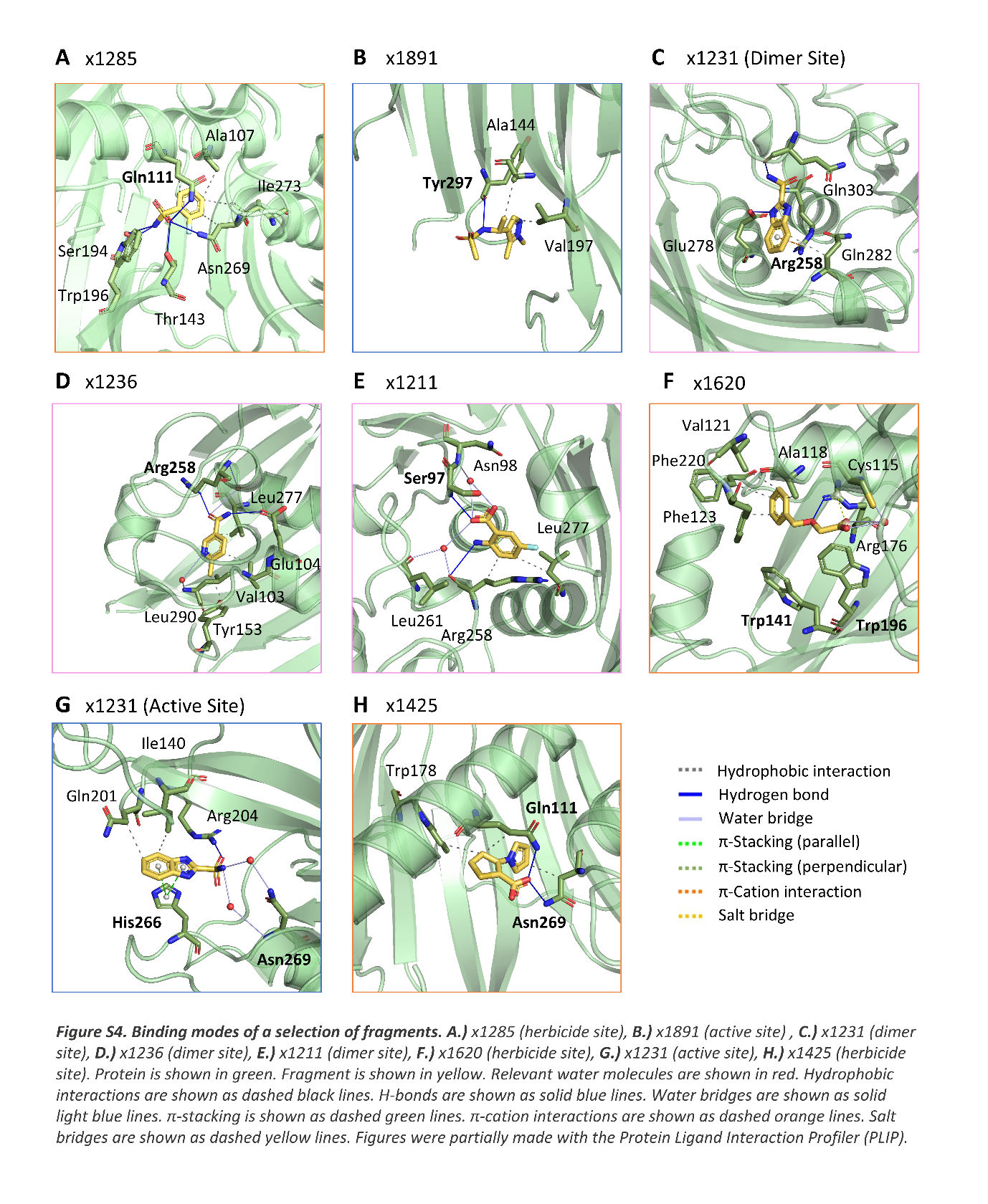


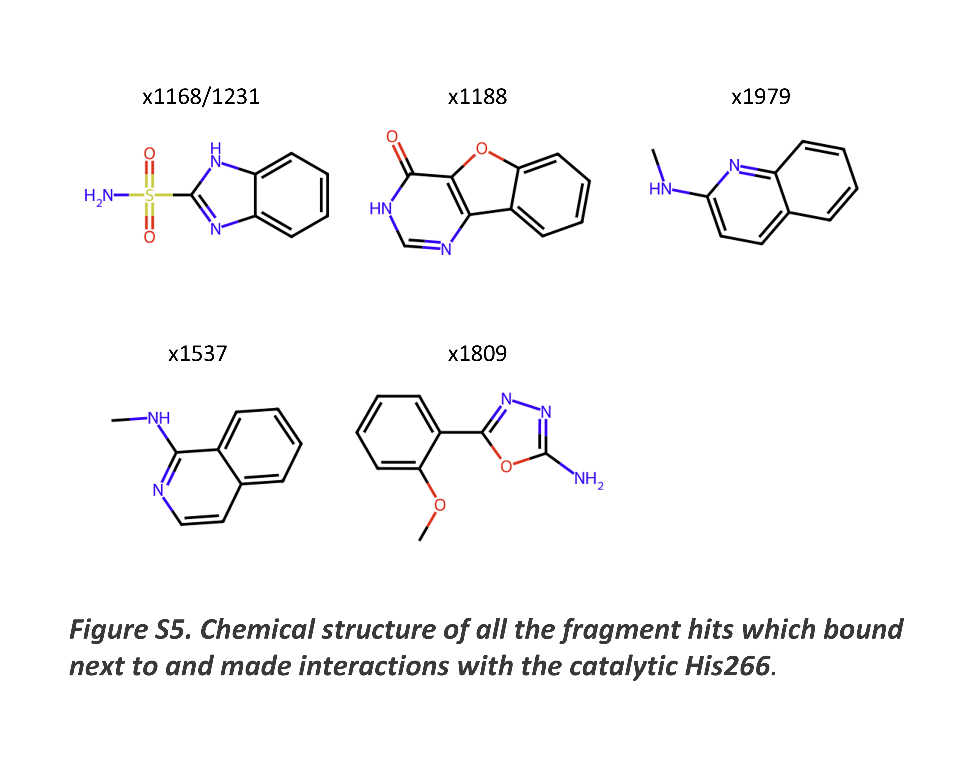


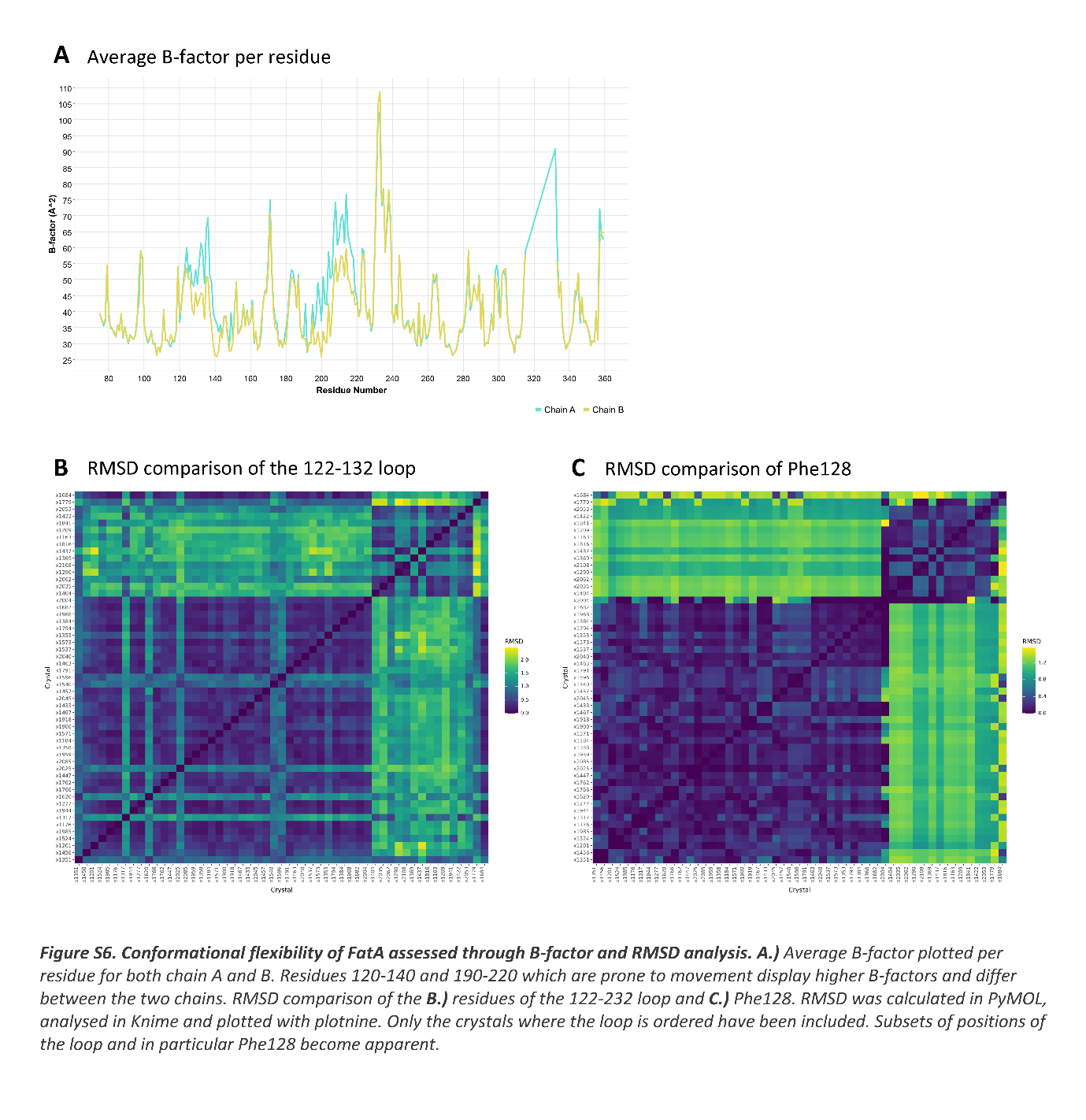


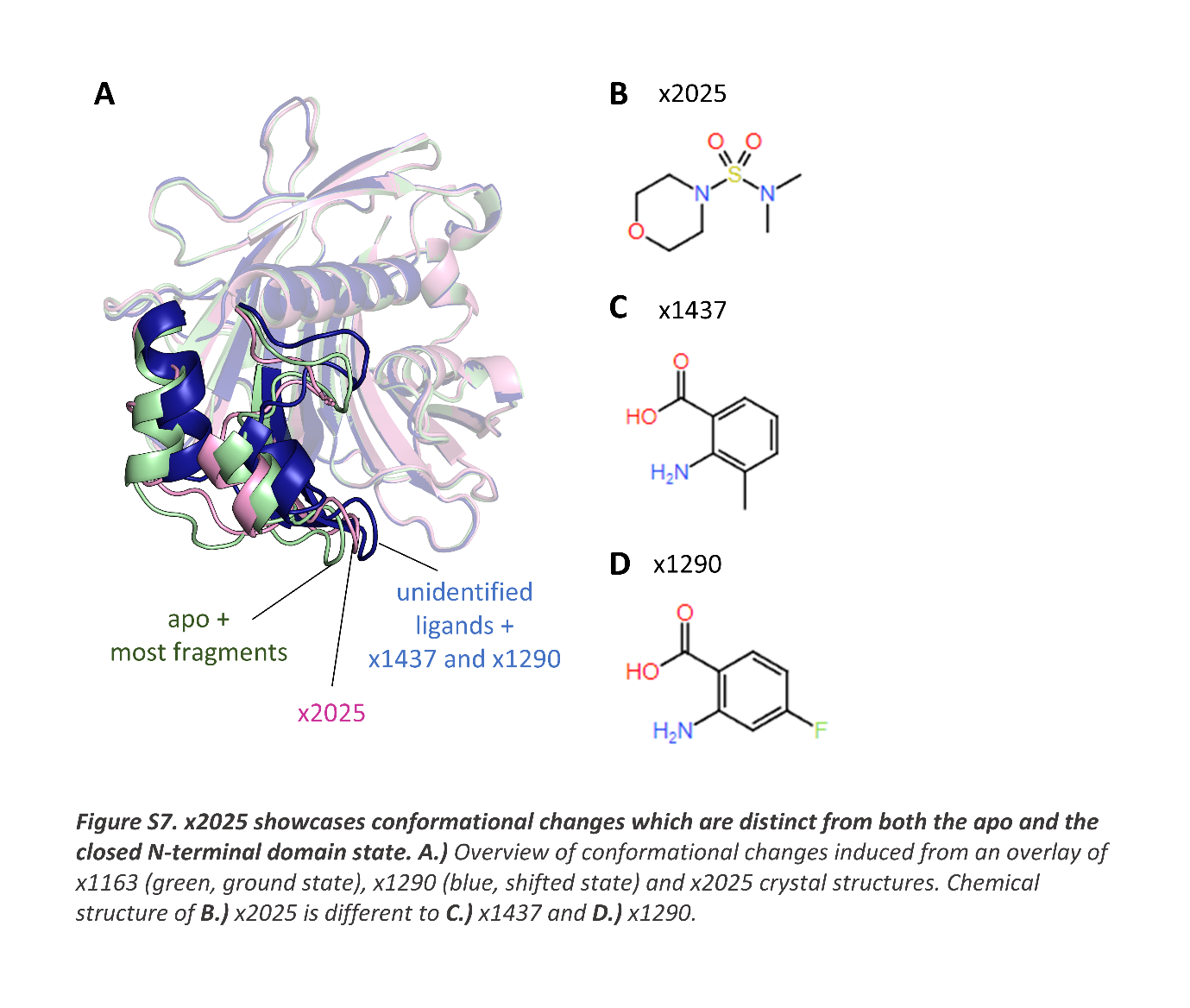


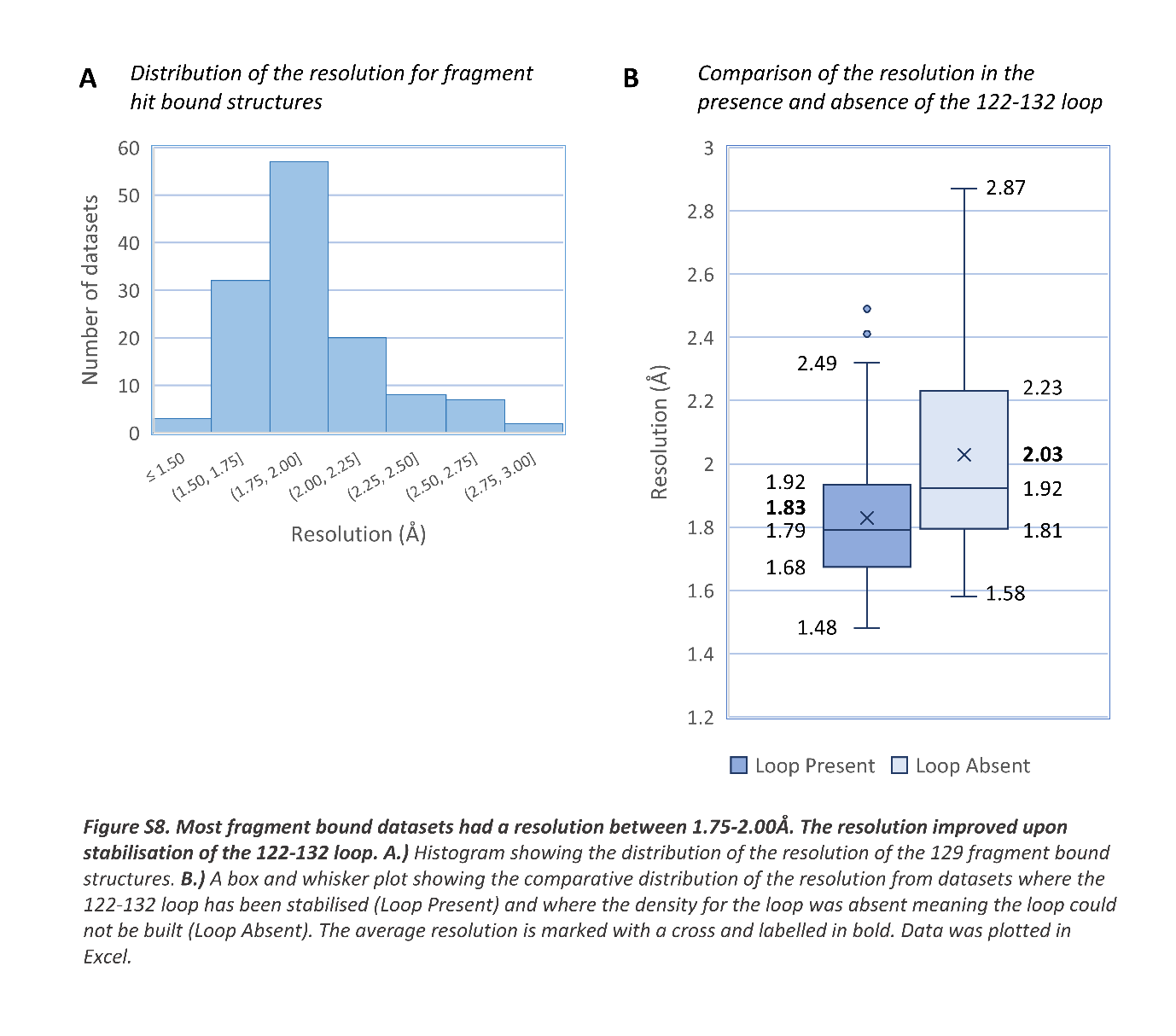


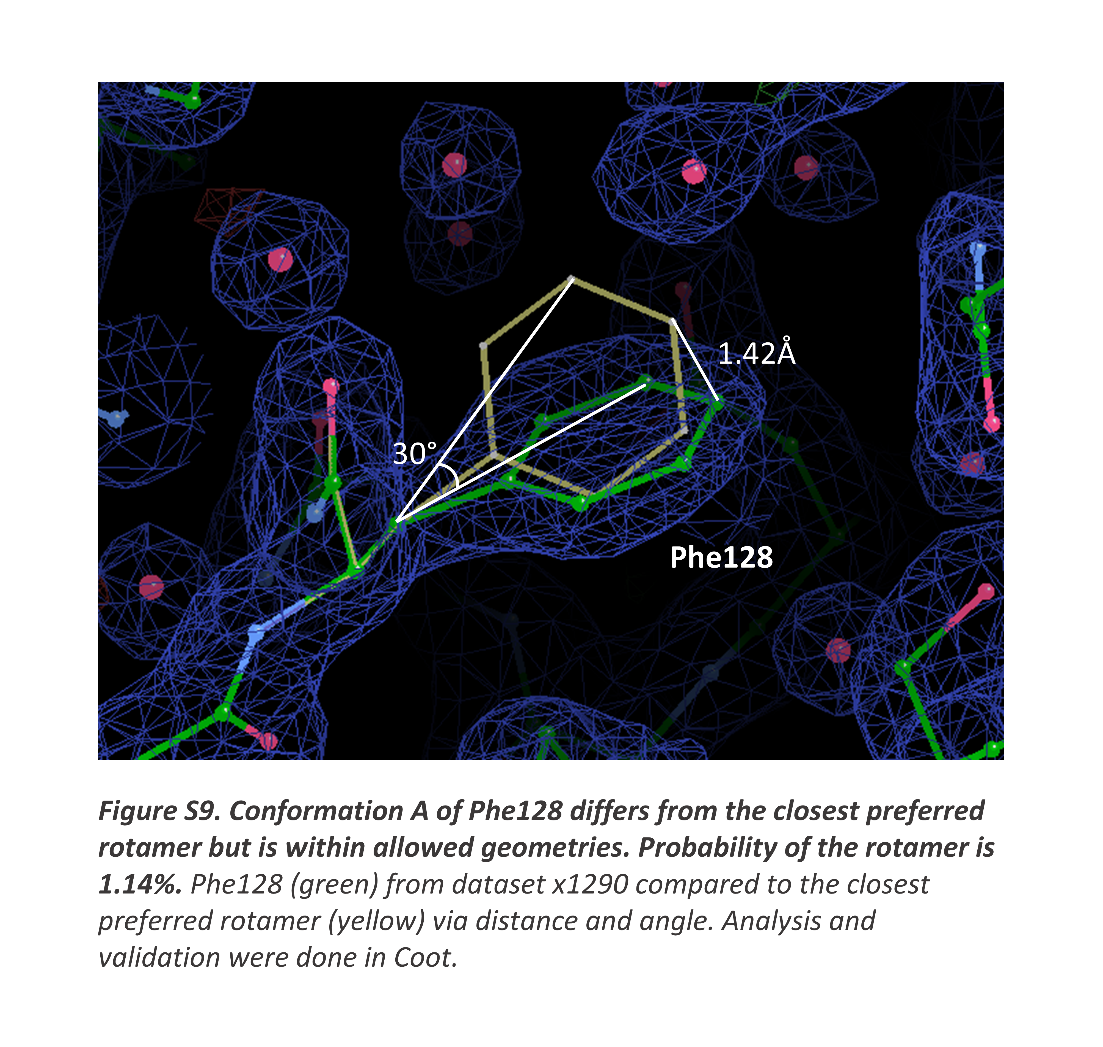


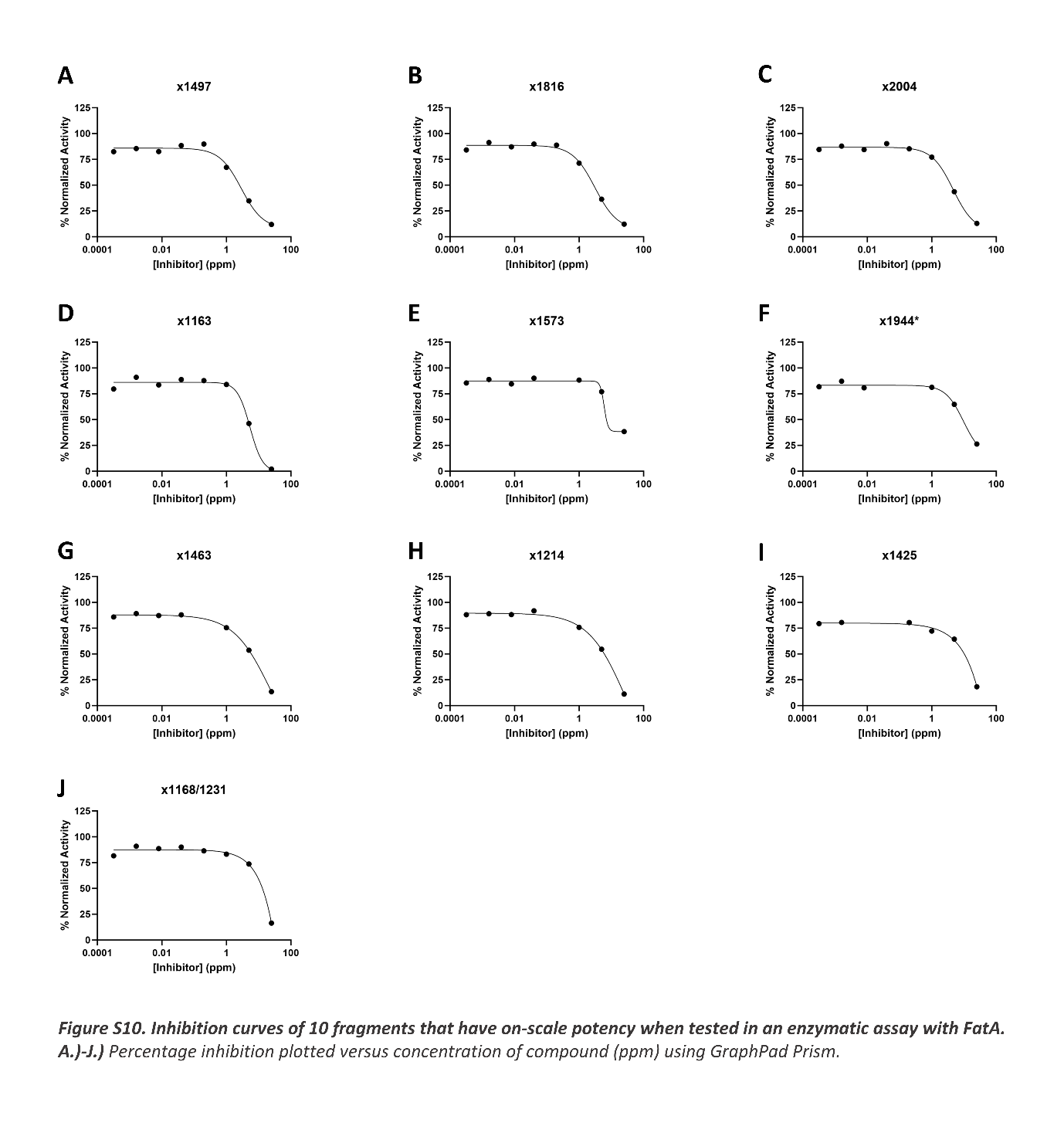


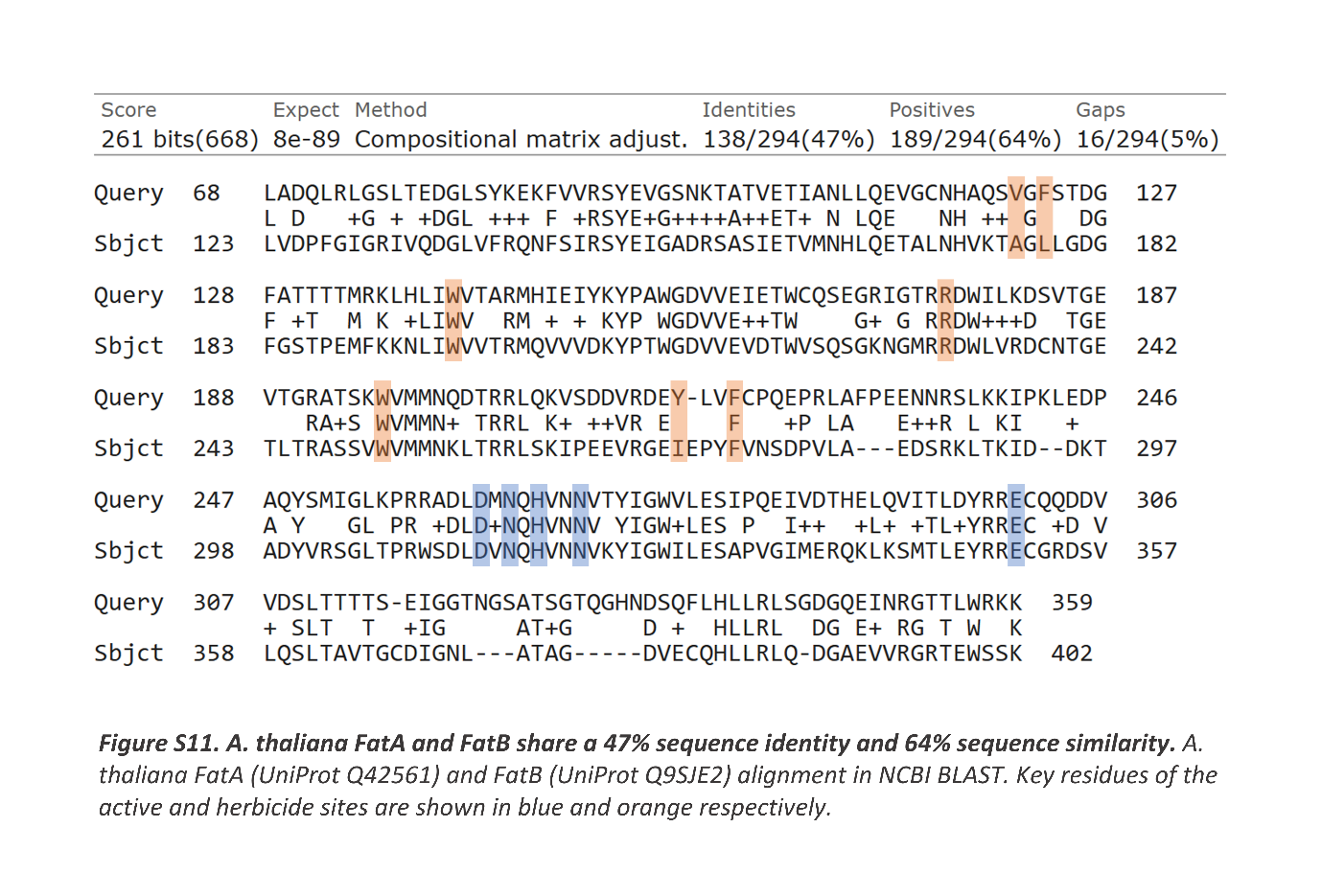

Supplement: Supplementary file 1 — Data S1. Supporting Information. [file PS-82-151-s002.docx]
